# From Chemical Scaffold to Predicted Toxicity: A Scaffold-Aware Pipeline for Reliable In Silico Prioritization of *Heracleum* Furanocoumarins

**DOI:** 10.3390/ijms27177947

**Published:** 2026-09-07

**Authors:** Anna E. Rassabina, Victor S. Safronov, Maxim V. Fedorov

**Affiliations:** 1 Institute for Information Transmission Problems, Russian Academy of Sciences (Kharkevich Institute), Bolshoy Karetny per. 19, Moscow 127051, Russia; safronov@phystech.edu (V.S.S.); fedorov@syntelly.com (M.V.F.); 2 ITMO University, 49 Kronverkskiy Pr., St. Petersburg 197101, Russia

**Keywords:** furanocoumarins, *Heracleum*, machine learning, artificial intelligence, toxicity

## Abstract

In silico methods generate numerous toxicity predictions for natural compounds, yet their mutual consistency and transferability to new structures are rarely verified, and the predictions are often taken at face value. Using furanocoumarins of the genus *Heracleum* as a model class of natural products, we developed a reproducible computational pipeline that evaluates not the predictions themselves but their reliability. Two datasets were analyzed: an extended reference set (DS1, 2008 compounds from PubChem) and a taxonomically verified natural set (DS2, 102 *Heracleum* compounds). The pipeline combined scaffold analysis, molecular docking against 44 off-target proteins, redocking, prediction of ten phenotypic toxicity endpoints, scaffold-resolved separability testing, and scaffold-aware machine learning. The chemical space was concentrated around two scaffolds, and the descriptor space was non-randomly organized with respect to them along all 13 axes (false discovery rate (FDR)-adjusted *p* < 0.01), indicating that the predicted toxicological profile is determined predominantly by molecular structure. The mean docking score corresponded to only the first principal component of the multidimensional binding profile, and the ten endpoints were largely independent of one another. Critically, scaffold separability did not predict transferability: structurally determined signals were only partially reproduced on unseen scaffolds, and to differing degrees across endpoints. A multi-criteria prioritization identified 21 top-priority candidates for experimental follow-up. The reliability of in silico assessments for this class cannot be assumed without scaffold-aware validation.

## 1. Introduction

Furanocoumarins of the family *Apiaceae*, genus *Heracleum*, are highly biologically active secondary metabolites [1,2,3]. These compounds represent a pharmacologically promising class: they are used in psoralen plus ultraviolet A (PUV-A) therapy [4] and in the development of anticancer and antimicrobial agents [5,6]. At the same time, furanocoumarins are toxic compounds that cause photodermatitis and chemical burns [7,8]. Their detailed characterization is therefore needed to assess the risks associated with their use across these applications.

The toxicity of the several hundred furanocoumarins known to date [9] is usually assessed experimentally through the determination of median lethal dose (*LD*_50_) values in various model organisms [4,10,11]. Such data, however, are available for only a few of them, since in vitro and in vivo studies are time-consuming and costly. Several well-established approaches to toxicity prediction have emerged to date [12]. Expert systems based on structural alerts [13,14] associate the presence of particular toxicophores with toxicity endpoints and underpin regulatory procedures, including the assessment of impurity mutagenicity under ICH M7 [15]. Classical quantitative structure–activity relationship (QSAR) and read-across, formalized in the OECD QSAR Toolbox [16], establish quantitative structure–activity relationships within a defined chemical series. The emergence of large curated datasets, primarily Tox21 and ToxCast [17,18], has made it possible to apply artificial intelligence (AI), and in particular machine learning (ML) models, on dozens of endpoints simultaneously, while graph neural networks and molecular representation learning methods (Chemprop, directed message-passing neural network (D-MPNN)-type architectures) have provided a further gain in performance over models built on fixed descriptors [19,20]. The same class of models has demonstrably accelerated drug discovery itself: generative deep learning applied to de novo molecular design has yielded structurally novel antibacterial compounds active against multidrug-resistant pathogens, including methicillin-resistant *Staphylococcus aureus* [21]. These developments underlie integrated web platforms for absorption, distribution, metabolism, excretion and toxicity (ADMET) prediction (pkCSM, ADMETlab, ProTox) [22,23,24], which combine dozens of models into a single toxicological profile. A separate line of work is associated with the adverse outcome pathway (AOP) concept [25], within which molecular docking is used to evaluate binding to a panel of protein targets as potential molecular initiating events (MIE) [26].

Building on our earlier analysis [27], we aimed to develop and validate a reproducible computational pipeline that assesses how consistently—in terms of structural determination—different in silico representations of a compound (molecular structure, off-target binding profile, and predicted toxicity) organize the chemical space of furanocoumarins, and whether this consistency enables robust prioritization of natural *Heracleum* compounds for further study. The key methodological question was whether the predicted toxicological profile is governed by independent biological information or by the molecular structure of the compound, and whether model-derived conclusions are transferable to new chemical scaffolds. The genus *Heracleum* serves as a model natural class on which the proposed analytical approach is demonstrated.

The pipeline was applied to two datasets of different nature: DS1, an extended reference set of the furanocoumarin chemotype (2008 compounds from PubChem, retrieved via psoralen and angelicin queries), and DS2, a taxonomically verified natural set from *Heracleum* (102 compounds). The pipeline sequentially comprised the following: mapping of structural diversity and scaffold analysis of molecular frameworks; visualization of the chemical space using t-SNE; molecular docking against a panel of 44 off-target proteins with analysis of the full binding profile; redocking; prediction of ten phenotypic toxicity endpoints and assessment of the scaffold-resolved organization of the descriptor space; evaluation of the transferability of structure–property models to new scaffolds and prioritization of the natural compounds of *Heracleum*.

## 2. Results

### 2.1. Dataset Formation

The DS1 dataset was assembled by retrieval from the PubChem database using two canonical furanocoumarin cores—psoralen and angelicin—and, after curation, contained 2008 compounds of natural and synthetic origin. We confirmed that according to the SMARTS-based scaffold classification, 1004 DS1 compounds belong to the linear psoralen-like type and 997 to the angular angelicin-like type, two molecules contain the psoralen and angelicin scaffolds, and a further five bear an additional benzofuran ring in addition to the furanocoumarin core. These categories are based on substructure matching and are not mutually exclusive, as a molecule may match more than one query. Both classical structural types are therefore well represented in DS1. DS1 was used as a reference space to position the natural compounds and, being restricted to two predefined structural queries, does not represent the exhaustive chemical space of coumarins or furanocoumarins.

The dataset retrieved from the “Furanocoumarins in *Apiaceae*” database covers 60 *Heracleum* species (74 taxa when infraspecific ranks are counted separately), which appear in the primary sources under 97 different names. After structure standardization, the union of DS1 and DS2 resulted in 2073 unique records, with 37 compounds being present in both sets, 1971 belonging to DS1 only, and 65 belonging to DS2 only. The chemotype composition of both datasets was determined by substructure matching against the linear (psoralen-type) and angular (angelicin-type) furanocoumarin cores and the 2H-chromen-2-one core. DS1 is essentially a pure furanocoumarin set: all but one of its 2008 compounds carry a fused furanocoumarin core, and only a single compound is a simple coumarin. DS2 is likewise furanocoumarin-dominated but retains a small non-fused fraction: 89 of the 102 compounds (87.3%) carry a furanocoumarin core (57 linear, 12 angular, 10 dihydro-linear, and 10 dihydro-angular types), 11 (10.8%) are simple coumarins, and two (2.0%) belong to neither class. The conclusions of this work therefore apply to the furanocoumarin chemical space and should not be extrapolated to simple coumarins, which are represented by a single compound in DS1 and by eleven compounds in DS2.

### 2.2. Structural Coverage of the Chemical Space

**Figure 1:**
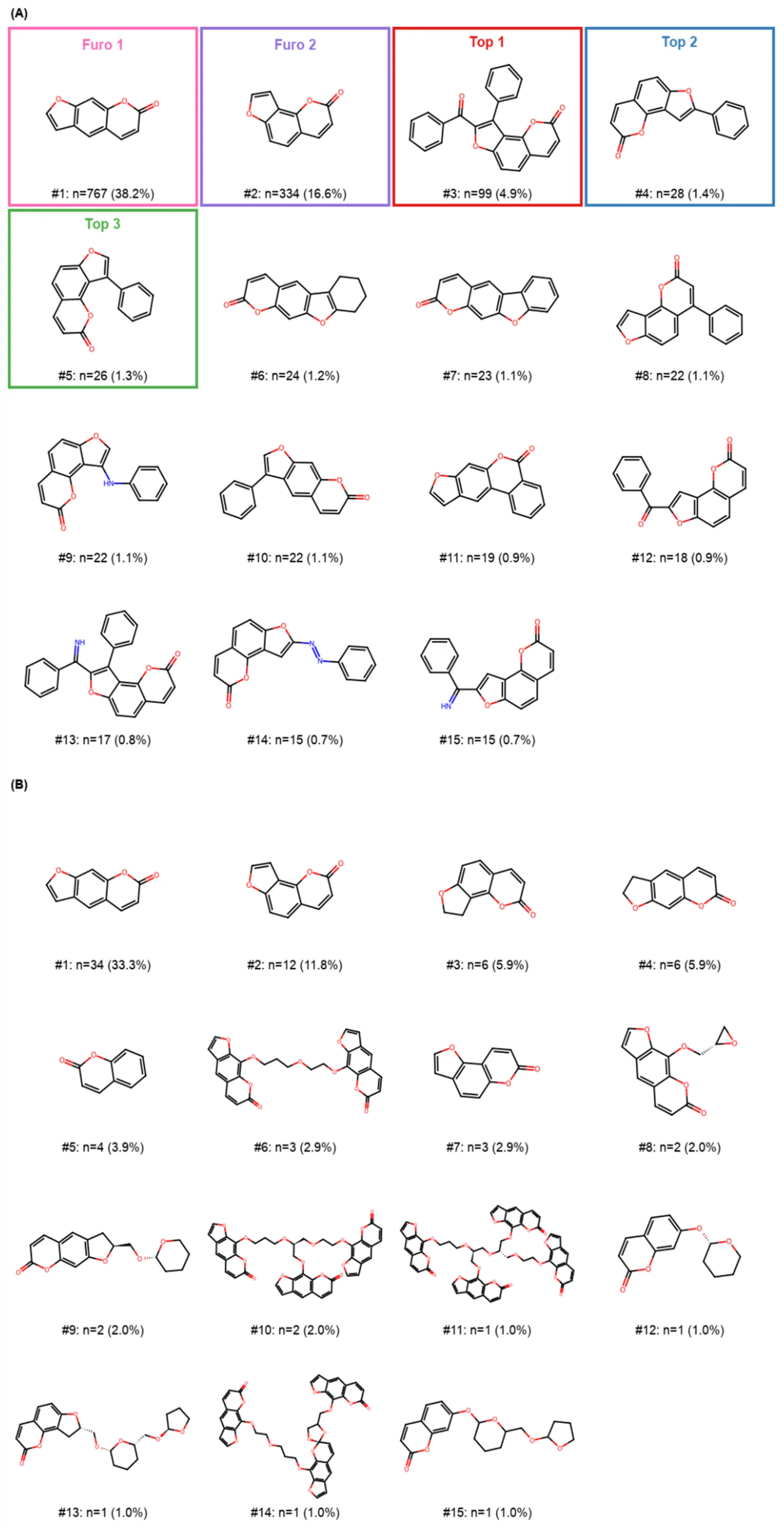
(**A**) Top 15 scaffolds in DS1 (2008 compounds) by occurrence frequency (*n*) and percentage distribution: #1 (Furo 1)—psoralen, #2 (Furo 2)—angelicin; #3 (Top 1), #12, #13, #15—benzoyl/iminobenzoyl derivatives bearing two phenyl groups (dicoumarol-like dimeric motifs or C-arylated structures); #4 (Top 2), #5 (Top 3), #8, #10—mono-phenyl-substituted psoralen/angelicin; #9, #14—N-phenyl and azo derivatives (aminated/diazotized cores); #6—saturated analogue (tetrahydro ring, CCCC4)—a partially reduced furanocoumarin; #7, #11—expanded/isomeric ring systems (pyranocoumarin or angularly extended core). (**B**) Top 15 scaffolds in the DS2 dataset by occurrence frequency (*n*) and percentage distribution: #1—psoralen, #2—angelicin, #3—angelicin analogue, #4—dihydropsoralen, #5—simple coumarin lacking a fused furan ring (umbelliferone-like type, unsubstituted benzopyranone core), #6, #10, #11—oligomeric ester conjugates of psoralen, #7—an isomeric ring-fusion system distinct from the classical psoralen and angelicin types (an alternative topology of furan–pyran ring fusion), #8—psoralen bearing an epoxide (oxirane) side substituent, #9, #13—dihydrofurocoumarins, #12, #15—a simple coumarin scaffold (as in #5) but bearing oxane/tetrahydrofuran ester substituents of varying complexity, #14—psoralen linked via an ester chain to a prenylated epoxy-pyran fragment and a second furocoumarin ring.

As a first step toward characterizing the structural features of the DS1 and DS2 datasets, we performed scaffold analysis. Two basic chemotypes were found to dominate both datasets (Figure 1A,B).

For 2008 compounds, we identified 330 unique Bemis–Murcko scaffolds and 201 generic scaffolds. For clarity, we present the top 15, from which we distinguish six scaffold classes (Figure 1A). Scaffold #1 (Furo 1, *n* = 767) corresponds to psoralen, the linear furanocoumarin core, while scaffold #2 (Furo 2, *n* = 334) corresponds to angelicin, the angular core. Together, these two scaffolds cover up to 54.8% of the DS1 dataset. There are 222 singletons (67.3% of scaffolds), and two scaffolds are needed to cover 50% of the molecules. We next consider Top 1 (*n* = 99), Top 2 (*n* = 28), and Top 3 (*n* = 26), as they stand out clearly against the “long tail” of the predominantly singleton scaffolds and, together with Furo 1 and Furo 2, account for 62.4% of the dataset—1254 of 2008 compounds. Most of the remaining scaffolds are psoralen/angelicin cores retaining an aryl substituent within the scaffold itself (Figure 1A). The subsequent structures #6–#15 are assigned to the “Other” category.

For the natural furanocoumarins (Figure 1B), we identified 38 unique Bemis–Murcko scaffolds and 30 unique generic scaffolds—the closeness of these two numbers indicates that the structural diversity of the set is determined primarily by the topology of the ring system (the number and type of fused rings, stereocenters, and additional fragments) rather than simply by atomic composition within an otherwise identical skeleton. The mean number of molecules per scaffold for DS2 is 2.68. Among the top 15, the dominant scaffold is psoralen (33%). As a result, more than 55% of the compounds in the dataset (58 of 102 compounds) reduce to just four basic frameworks, covering the two classical structural types of furanocoumarins—linear and angular—each in aromatic and dihydro form. Another 4% of the compounds fall within a single scaffold corresponding to a simple coumarin lacking a fused furan ring; simple coumarins in DS2 are not confined to this scaffold and amount to 11 compounds in total (Section 2.1). Overall, the top 15 scaffolds cover 79 of 102 compounds (77.5%), while the remainder are distributed across 23 unique singleton scaffolds. Only three scaffolds are needed to cover 50% of the molecules.

The position of the natural DS2 compounds within the DS1 chemical space is shown in Supplementary Materials Figure S1. The bulk of the compounds is localized within a single region, indicating a high degree of structural homogeneity for a substantial portion of the set. Beyond this, a gradual structural divergence is observed—compounds increasingly deviate from the core skeleton with distance from it without forming a sharply separated cluster. A subset of the red points is scattered along this zone of structural divergence, corresponding to the few natural analogs that deviate from the main skeleton.

### 2.3. Off-Target Binding Landscape: Full Profile Versus Integrative Metric

#### 2.3.1. Principal Component Analysis (PCA)

Molecular docking of 2055 ligands to the binding sites of 44 off-target proteins (Supplementary Materials Table S1a–c) was performed using AutoDock Vina 1.2.5. The negative controls, selected on the basis of documented absence of binding, covered all 44 targets of the panel and included mescaline for the mu-opioid receptor (OPRM1, −5.2 kcal/mol), psilocin for the GABA-A receptor (GABRA1, −7.7 kcal/mol), thymol for the Cav1.2 calcium channel (CACNA1C, −5.6 kcal/mol), and N-2-naphthylanthranilic acid for the androgen receptor (AR, −9.5 kcal/mol). Their median score across the panel was −7.3 kcal/mol, compared with −7.8 for known active ligands. At the level of individual targets, active ligands docked significantly more favorably than inactive ones for only 13 of the 39 targets, with a sufficient number of observations (Mann–Whitney test and Bonferroni correction).

**Figure 2:**
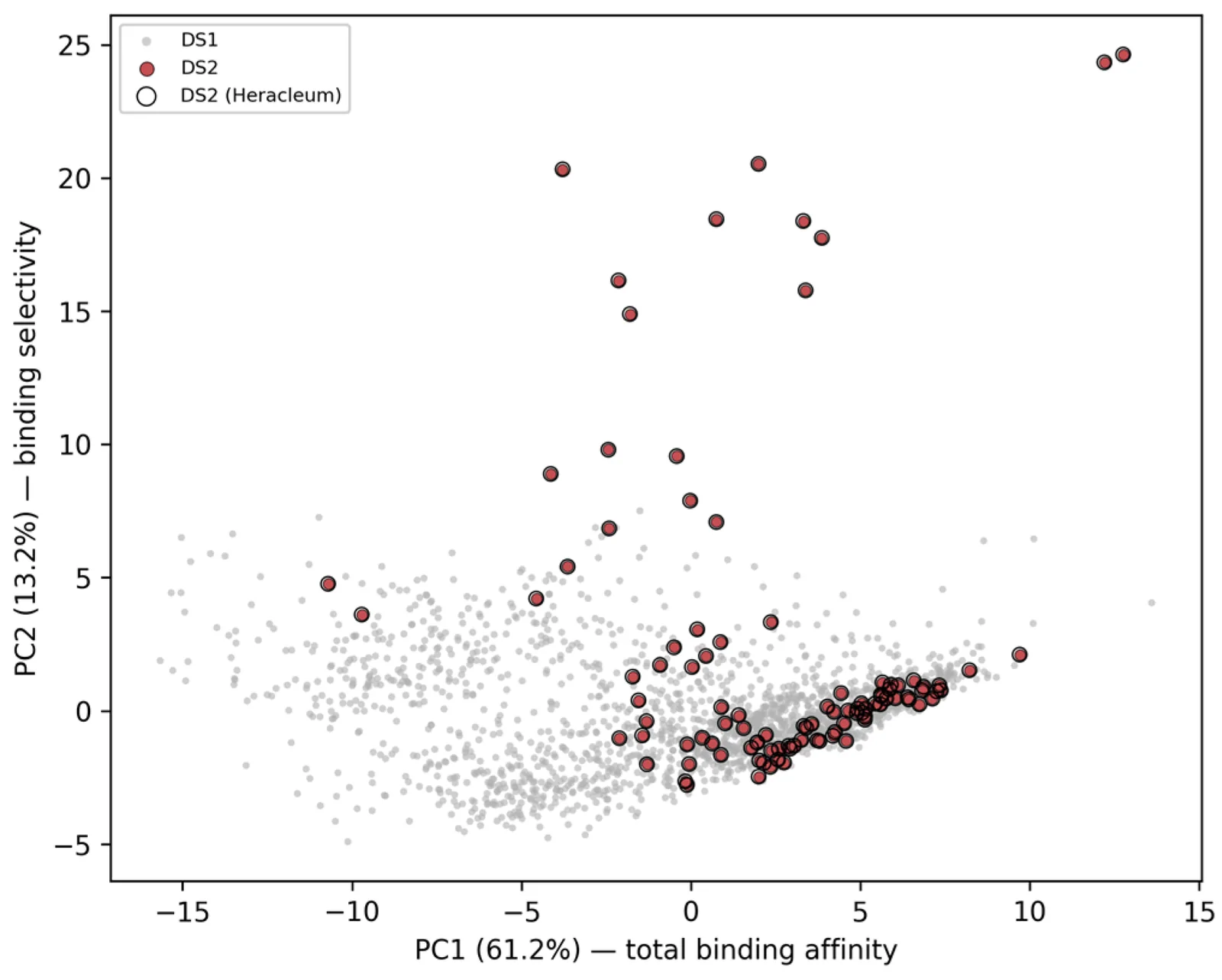
Principal component analysis (PCA) of the full off-target binding profile for 2055 ligands with a valid 44-dimensional docking profile. Projection of the compounds onto the first two principal components: PC1 (61.2% of variance) corresponds to overall binding affinity (ρ = 0.927 with the mean docking score across the 44 targets; higher values indicate weaker binding), and PC2 (13.2%) to binding selectivity. Compounds of the reference set DS1 are shown in grey, and the 102 natural furanocoumarins of the genus *Heracleum* (DS2) in red with a black outline.

To characterize the structure of the full off-target binding profile to establish how many independent axes of variation it contains and which targets define them, we applied principal component analysis (PCA) to a 2055 × 44 docking score (Figure 2).

The first principal component explained 61.2% of the variance; the second explained 13.2%; and the third and fourth explained 3.3% and 2.5%, respectively. Four components were required to describe 80% of the variance (Supplementary Materials Figure S2). It is worth noting that we had also previously computed the mean docking score across the 44 targets (Supplementary Materials Figure S3). The first component coincided almost entirely with the mean docking score across the 44 targets (ρ = 0.927, Spearman) (Supplementary Materials Figure S4), which shows that averaging the profile captures precisely this largest axis of variation—the overall binding affinity—but ignores the subsequent components. Thus, the second component (13.2% of variance) was determined predominantly by loadings on PTGS2, HRH2, ADRA1A, MAOA, and AR, and it reflected not the strength but rather the selectivity of binding—a shift of the profile toward the inflammatory–adrenergic subgroup of targets. The third component separated molecules by their binding to histamine (HRH1) and muscarinic (CHRM2) receptors. Thus, the true structure of off-target binding is at least four-dimensional, and reducing it to a mean value eliminates the interpretable axes of selectivity.

The fourth component contributed to reaching the 80% threshold but had no clear interpretation regarding distinct target subgroups and was not considered further. To quantitatively characterize the breadth of binding, promiscuity descriptors were computed (Supplementary Materials Figure S5). The number of targets with strong predicted binding (docking score < −10 kcal/mol) ranged from 0 to 29, with a median of 1 and a mean of 5.6, indicating a pronounced right-skewed distribution: most compounds are selective, while a small fraction exhibits a broad promiscuous profile. The natural *Heracleum* compounds (DS2) were characterized, on average, by a weaker and less promiscuous binding profile than the extended reference set: the median PC1 for the 37 natural compounds overlapping with DS1 was 4.19 versus 1.14 for the DS1-only compounds (higher PC1 values correspond to weaker binding), and their median number of strongly bound targets was zero. This is consistent with the notion that natural *Heracleum* furanocoumarins are not high-affinity promiscuous ligands but rather occupy a region of moderate, selective binding.

#### 2.3.2. Redocking Experiments

To validate the docking protocol, the co-crystallized ligands were re-docked into 42 structures of the panel using the same AutoDock Vina settings as in the main calculation (Supplementary Materials Table S1a,b). Symmetry-aware heavy-atom RMSD was computed in the fixed receptor coordinate frame without structural superposition. The top-ranked pose reproduced the crystallographic pose within 2.0 Å for 24 of 42 complexes (57.1%). When the five top-ranked poses were considered, a near-native conformation was recovered for 36 of 42 complexes (85.7%). The median RMSD was 0.83 Å. The six cases of unsuccessful recovery (HRH2, HTR1A, CACNA1C, SCN5A, PTGS1 and MAOA; RMSD 2.74–7.07 Å) correspond to three system types: small polar ligands in shallow, solvent-exposed sites (histamine in the H2 receptor); large flexible ligands in the wide pores of ion channels (quinidine in Nav1.5, sofosbuvir in Cav1.2); and ligands whose crystallographic binding mode depends on induced fit or on an environment not reproduced by the rigid receptor model (ST171 in 5-HT1A, celecoxib in the COX-1 side pocket, and harmine in the FAD-containing site of MAO-A). Docking-derived conclusions for these six targets were interpreted with caution.

### 2.4. Predicted Toxicity Landscape

**Figure 3:**
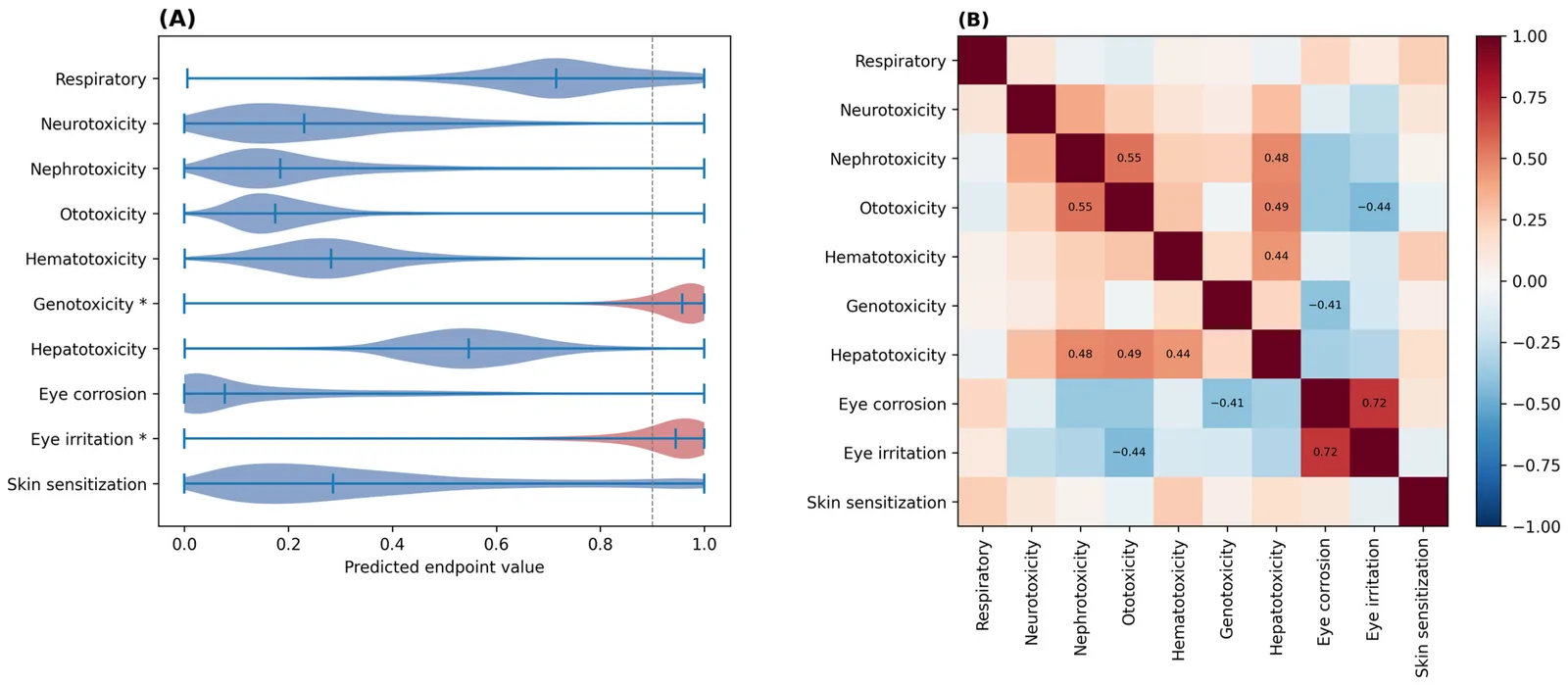
(**A**) Violin plots of the predicted value distributions for the ten endpoints. Asterisks (*) mark saturated endpoints—those whose distribution is concentrated near the upper bound (dashed grey line) and which therefore provide little discriminative power; these are also shown in red. Vertical bars denote medians and the range of the data. (**B**) Correlation matrix (Spearman) with two blocks highlighted.

For the ten phenotypic toxicity endpoints predicted by ADMETLab 3.0—respiratory, neurotoxicity, nephrotoxicity, ototoxicity, hematotoxicity, genotoxicity, hepatotoxicity, eye corrosion, eye irritation, and skin sensitization—the distributions of each endpoint, their cross-correlations, and how many independent axes in this space (PCA) were analyzed for all 2073 compounds in the combined DS1 and DS2 set (Figure 3A).

The resolving power of the endpoints was markedly heterogeneous. Two endpoints proved to be saturated at the upper bound of the scale and virtually failed to discriminate between compounds: genotoxicity (median 0.958; 74.8% of compounds with a value >0.9) and eye irritation (median 0.945; 65.5% > 0.9) (Supplementary Materials Table S2). The eye corrosion endpoint, by contrast, was shifted toward the lower bound (54% of values <0.1) but retained a broad spread (IQR = 0.28). The highest resolving power (by interquartile range) was shown by skin sensitization (IQR = 0.34), eye corrosion (0.28), neurotoxicity (0.25), and respiratory (0.19). The saturated endpoints (genotoxicity and eye irritation) were excluded from the subsequent ranking of compounds as uninformative but were retained in the descriptive analysis.

The endpoints formed interpretable correlated clusters. Correlation analysis (Spearman) revealed two pronounced blocks (Figure 3B). The first grouped organ toxicity measures nephrotoxicity, ototoxicity, hepatotoxicity, and hematotoxicity (pairwise ρ from 0.44 to 0.55). The second linked the two ocular endpoints: eye corrosion and eye irritation (ρ = 0.72). The presence of such blocks indicates that some of the ten endpoints carry overlapping information and confirms the need for dimensionality reduction prior to integrative interpretation (Figure 3B).

The toxicity space is weakly compressible, unlike the docking space. PCA of the ten endpoints showed that the first component explains only 29.6% of the variance, and six components are required to describe 80% (Supplementary Materials Figure S6). This differs fundamentally from the off-target binding space (Section 2.3), where a single component covers 61% of the variance. In other words, the predicted toxicological endpoints are largely independent of one another and do not reduce to a single axis of “overall toxicity”: a compound may be favorable on some endpoints and unfavorable on others. This result justifies abandoning any single integrative toxicity index and confirms the validity of the per-endpoint analysis in the subsequent sections.

### 2.5. Scaffold-Resolved Organization of the Descriptor Space

**Figure 4:**
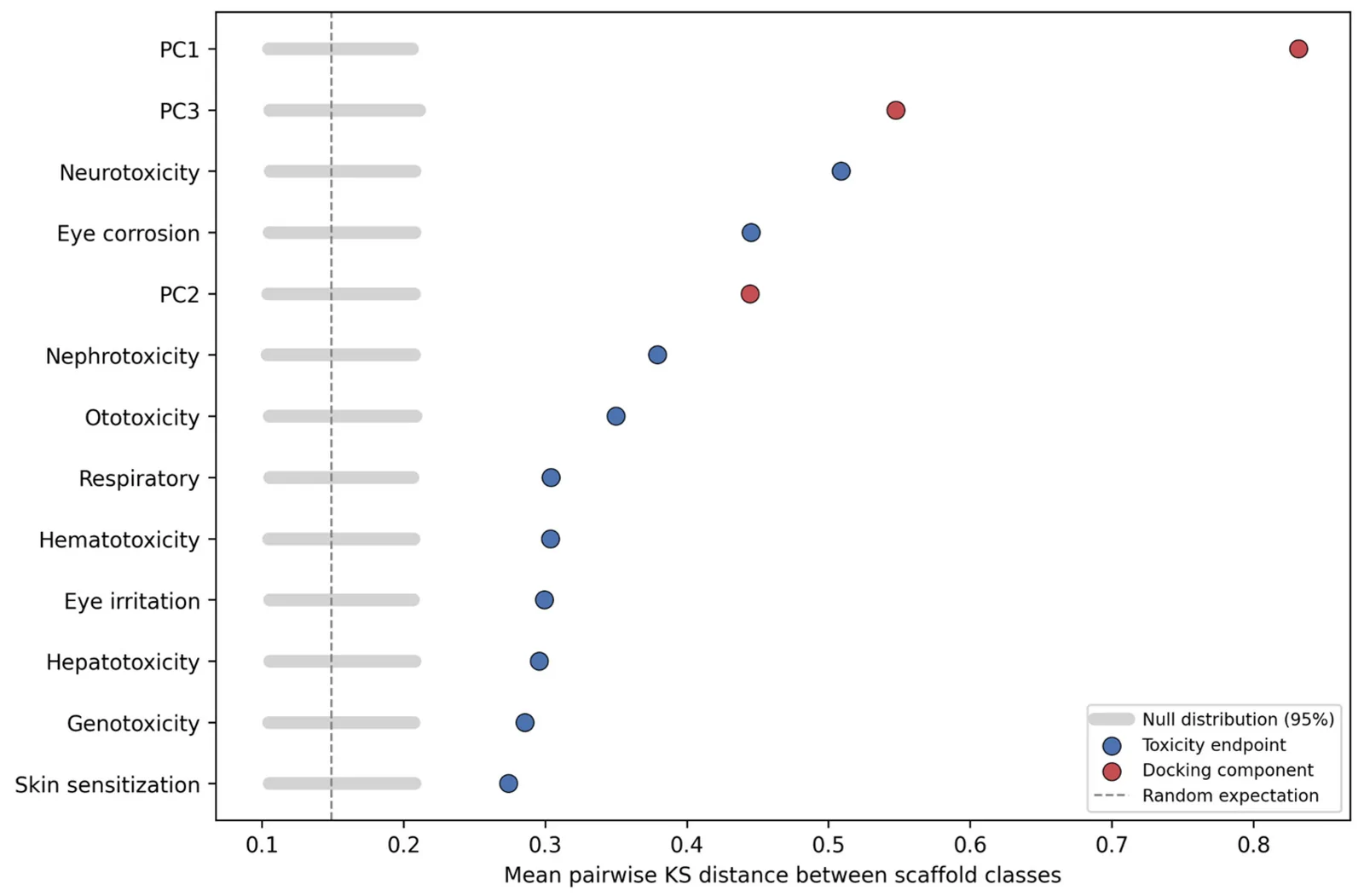
Results of the permutation test: for each of the 13 axes, the observed KS (point/bar) against the null distribution. The axes are sorted by observed KS. The dashed line indicates the level of random expectation (~0.149). 10,000 permutations, all p_FDR < 0.01.

Having characterized the structure of the off-target binding and predicted phenotypic toxicity spaces separately, we tested whether these spaces are organized non-randomly with respect to the chemical structure of the compounds (Figure 4). As the scaffold class and all descriptors (docking and ADMET) are structure-derived quantities, this analysis was not regarded as an external biological validation of the predicted toxicity. Its aim was to test chemical coherence: whether the computed descriptor space retains a non-random organization with respect to the chemically defined scaffold classes. If the descriptor space were arbitrary with respect to the molecular structure, the scaffold class labels would not yield separability above random expectation. Thus, the test assessed not the truth of in vivo toxicity but rather the interpretability of the descriptor landscape and the suitability of the scaffold classes as a structural layer for selecting subsequent experimental hypotheses.

We tested whether the scaffold classes Furo 1, Furo 2, and Top 1–3 differ across 13 axes—the ten predicted toxicity endpoints (Section 2.4) and the first three principal components of the off-target binding profile PC1–PC3 (Section 2.3)—and, critically, whether this difference is non-random (Figure 4).

For a given feature, all class pairs were taken (Furo 1 vs. Furo 2, Furo 1 vs. Top 1, etc., with 10 pairs from 5 groups), and, for each pair, the KS statistic (Kolmogorov–Smirnov test) was computed, which indicates the degree to which the distributions of that feature differ between the two classes (0 = identical and 1 = completely separated) (Supplementary Materials Table S6a–d).

Furo 1 and Furo 2 are the prevalent scaffolds, as we are dealing with furanocoumarins. Top 2 and Top 3 are positional isomers of the angelicin core bearing a single phenyl substituent on the furan ring; Top 1 is a more structurally elaborated variant carrying a phenyl and a benzoyl group. The Other class, which groups rare scaffolds, was excluded from the statistical test because it did not constitute a distinct structural class (Figure 1).

For each axis, the mean pairwise KS distance between the five scaffold classes was computed; significance was assessed using a permutation test (10,000 permutations of the labels while preserving group sizes, seed = 42) with correction for multiple comparisons (Benjamini–Hochberg).

The observed scaffold separability exceeded the random expectation along all 13 axes (p_FDR < 0.01). The mean pairwise KS distance under random label permutation was approximately 0.149 for all axes, whereas the observed values were substantially higher. The mean across the ten toxicity endpoints was 0.345 versus 0.149 under random permutation.

The strongest scaffold-associated separability was shown by the first principal component of the docking profile (PC1, KS = 0.832), indicating a pronounced structural determination of the overall binding affinity. The third and second components also exhibited high separability (PC3 = 0.548; PC2 = 0.445), meaning that the scaffold classes differ not only in the strength but also the selectivity of binding. Replacing the integrative metric “mean 44” with the components of the full profile increased the resolution of the test: PC1 alone separated the classes markedly more strongly than the previously used mean docking score (0.599) (Supplementary Materials Table S5).

Among the toxicity endpoints, the highest scaffold-associated separability was shown by neurotoxicity (0.509), eye corrosion (0.445), nephrotoxicity (0.379), and ototoxicity (0.350). These axes represent the most informative directions for subsequent experimental and mechanistic studies: the docking profile components as axes for selecting off-target hypotheses, and neurotoxicity, eye corrosion, nephrotoxicity, and ototoxicity as priority endpoints for follow-up validation. The saturated endpoints (genotoxicity and eye irritation; Section 2.4) showed moderate separability (0.286 and 0.300) but, owing to scale saturation, are of limited use for ranking. Despite the high scaffold separability along PC1, the dominant natural scaffolds Furo 1/2 did not shift toward the region of the strongest binding.

### 2.6. Scaffold-Aware ML

The structural determination of the descriptor space established in Section 2.3, Section 2.4 and Section 2.5 raises the question of transferability: does a model trained to predict toxicity from structure retain its performance on compounds whose scaffold was not represented in the training set (Figure 1B)? In this section, we assess whether predictions can be trusted when the model encounters skeletons dissimilar to those in the training data.

**Figure 5:**
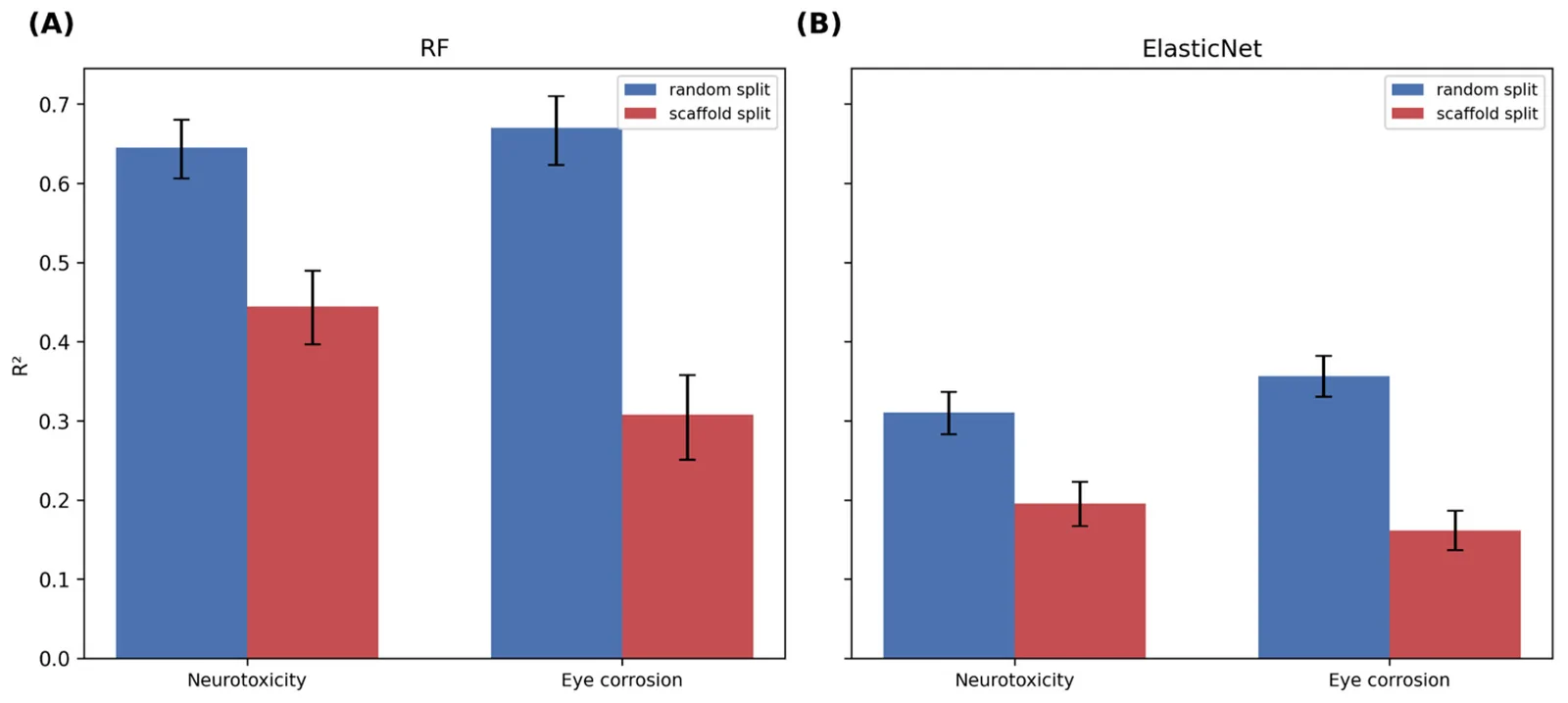
Transferability of the structure-property signal: random split versus scaffold split. Grouped bar plots of R^2^ (bootstrap 95% confidence intervals) for two toxicity endpoints (neurotoxicity, eye corrosion) under random-split (blue) and scaffold-split (red) cross-validation. (**A**) Random Forest (RF); (**B**) Elastic Net. Across both models, scaffold splitting reduces R^2^ relative to random splitting, and the decline is greater for eye corrosion than for neurotoxicity.

We trained Elastic Net and Random Forest models on Morgan fingerprints under two cross-validation schemes—random split and scaffold split (GroupKFold by the generic Bemis–Murcko scaffold)—and compared performance based on the generalization gap for two non-saturated endpoints with high scaffold separability: neurotoxicity and eye corrosion (Figure 5A,B).

Model performance is summarized in Supplementary Materials Table S3a,b, which reports R^2^, RMSE, and MAE for both endpoints under random and scaffold-based five-fold cross-validation. Both endpoints are scored on a 0–1 scale; thus, the RMSE and MAE are directly interpretable as absolute errors in the units of the endpoint. Enforcing scaffold-disjoint folds degrades every model–endpoint combination: for the Random Forest, R^2^ falls from 0.645 to 0.444 for neurotoxicity and from 0.670 to 0.308 for eye corrosion, while the RMSE rises by 25% and 45% and the MAE by 31% and 50%; as a result, the residual error under scaffold splitting reaches 0.75 and 0.83 of the target’s own standard deviation. The magnitude of the decline, however, differs substantially between endpoints: for the Random Forest, the generalization gap is moderate for neurotoxicity (ΔR^2^ = 0.200) but nearly twice as large for eye corrosion (ΔR^2^ = 0.362), where the residual error under scaffold splitting approaches the intrinsic spread of the endpoint, and little practical predictive value remains outside the training scaffolds. This indicates that part of the apparently high performance under standard random validation stems from the structural similarity between the test and training compounds rather than from a transferable structure–property signal, and that the degree of true generalizability differs between endpoints, even with comparable scaffold separability (Section 2.5).

### 2.7. Tier-Based Prioritization of Heracleum Compounds

The method developed in Section 2.3, Section 2.4, Section 2.5 and Section 2.6 was applied to a practical task: the selection of natural *Heracleum* compounds. Given the limited transferability of the model predictions established in Section 2.6, prioritization was based not on a single integrative metric but on several independent, interpretable criteria with division into tiers, allowing the basis for selecting each compound to be traced transparently.

For the 102 natural DS2 compounds, the full off-target binding profile, the 10 predicted toxicity endpoints, and two sets of structural filters—PAINS (Pan-Assay INterference compoundS) and Brenk—were available. For further analysis, among the 10 endpoints, 2 saturated ones were removed—genotoxicity and eye irritation. For each of the eight informative endpoints, the median across all 102 compounds was computed. A compound was assigned to the priority set when all criteria were satisfied simultaneously (Section 4.7). PAINS were found to be zero for all 102 compounds; none of the furanocoumarins contained such fragments.

A favorable profile is defined as predicted toxicity below the set median across most of the informative endpoints, together with low promiscuity and the absence of structural alerts. Based on these criteria, 21 of the 102 compounds were assigned to the first priority tier, 24 to the second, and 57 to the third. Among the first-tier (Tier 1) priority compounds are heratomol, xanthotoxin, and oroselol, characterized by a favorable predicted profile across most endpoints (Supplementary Materials Table S4), low off-target promiscuity, and the absence of PAINS and Brenk structural alerts. The compound pabularinone was favorable across all eight informative endpoints. The 21 first-tier compounds include xanthotoxin and bergapten, which are already used in clinical practice. A comparison of the selected compounds with published experimental data is presented in Section 3. For clarity, the 2D structures of the 21 priority compounds are provided in Supplementary Materials Figure S7.

## 3. Discussion

When developing computational prioritization strategies for chemical compounds, one is confronted with the fact that in silico methods generate numerous toxicological predictions whose mutual consistency and transferability to novel structures are rarely verified, while the predictions themselves are often accepted as a finished result [28,29]. The present work is devoted to assessing the consistency and reproducibility of different in silico representations in organizing the chemical space of furanocoumarins from 60 species of the genus *Heracleum*. Furanocoumarins are of practical significance, and it is important to conduct in silico evaluations of the risks associated with their toxicity and to assess their safety. Natural furanocoumarins of the genus *Heracleum*, designated DS2, are distributed unevenly across the chemical space: most of them are localized within a dense core, which indicates the structural similarity between most known natural compounds and the dominant linear scaffold type (Supplementary Materials Figure S1). The linear psoralen and angular angelicin cores cover more than half of both the reference set DS1 and the natural set DS2 (Figure 1A,B). Among the Bemis–Murcko scaffolds of DS1, the ratio of linear (Furo 1) to angular (Furo 2) cores was 2.3:1; thus, linear psoralens predominate, which is consistent with the known chemotaxonomy of *Apiaceae* [30,31]. Structural diversity within this class is therefore realized mainly through peripheral modifications rather than through the emergence of new ring systems. The structural diversity of DS2 arises from peripheral functionalization of two core scaffolds: O-methylation and hydroxylation at C-5 and C-8, O-prenylation and geranylation (imperatorin, phellopterin, and bergamottin), epoxidation of the prenyl side chain followed by hydrolysis to the diol (oxypeucedanin and heraclenol), and esterification or glycosylation of the resulting hydroxyl groups.

Among the in silico approaches that allow for the modeling of MIE, molecular docking is a promising tool for toxicity screening, in which the molecular mechanisms of toxicity are modeled on the basis of the structure of the ligand–receptor complex and the degree of binding affinity [26]. Molecular docking was applied, for example, to identify MIE by Jeong (2023) [26]. Here, we instead used binding affinities as a descriptor space (Figure 2).

Analysis of the complete binding profile showed that the widely used mean docking score accounted for 61% of the variance and reflected the overall binding affinity but ignored independent axes of selectivity. The first component proved to be the most strongly structure-determined, with a scaffold separability of KS = 0.832, markedly higher than that of the averaged measure. The second and third components, associated with specific target subgroups, also separated scaffold classes significantly. This means that structure determines not only the strength but also the selectivity of off-target binding. The space of predicted phenotypic toxicity, by contrast, proved poorly compressible: the ten endpoints were largely independent, requiring six principal components to explain 80% of the variance, and a single integral toxicity index is therefore uninformative for this class (Supplementary Materials Figure S6).

These observations justify abandoning both the averaged docking score and a summary toxicity index in favor of a multidimensional, endpoint-wise analysis. Two saturated endpoints are also unsuitable for ranking.

The key result is that the descriptor space, both of off-target binding and predicted toxicity, is organized non-randomly with respect to scaffold classes: the observed separability exceeded random expectation along all 13 axes at p_FDR < 0.01 (Figure 4). It is essential to note that this test does not constitute external biological validation, as scaffold class, docking scores, and ADMET predictions are all quantities derived from structure. High agreement between them therefore reflects not biological truth but chemical coherence, that is, the fact that different structurally derived representations place the molecule in a consistent local environment. This increases the reproducibility of the computational hypothesis but does not confirm toxicity in vivo. This distinction is fundamental and differentiates the proposed approach from that in studies where agreement between several in silico models is erroneously interpreted as their mutual validation.

A practically significant result concerns the transferability of predictions. In computational toxicology and studies of natural compounds, ADMET model predictions are frequently evaluated using random data splits and are accepted as applicable to novel compounds. Our results show, however, that for the furanocoumarin class, such an evaluation systematically overestimates the true generalization capacity: under scaffold-based splitting, predictive quality declined for all endpoints considered (Supplementary Materials Table S3a), meaning that part of the accuracy obtained under standard validation was provided by the structural similarity between the test and training compounds rather than by a transferable structure–function signal. Moreover, the degree of transferability differed between endpoints with comparable scaffold separability: neurotoxicity retained moderate generalizability to new scaffolds, whereas eye corrosion lost it to a considerably greater extent. This leads to a non-trivial conclusion: scaffold separability and transferability are not equivalent. High structural determination of an endpoint indicates only that it is organized with respect to the scaffold but does not guarantee that a model will reproduce this signal on scaffolds absent from training; a formally informative endpoint may rely predominantly on the recognition of familiar structural classes. Assessment of scaffold separability is therefore insufficient in itself for judging predictive reliability, and analysis of the generalization gap is required as a separate stage in the interpretation of in silico results for this class. Eye corrosion is one of the most scaffold-separable endpoints. Furanocoumarins are clinically known for their capacity to cause phytophotodermatitis and damage to the skin and eyes upon contact under ultraviolet (UV) irradiation [32,33]. The fact that descriptor analysis independently singled out this particular endpoint as structurally determined resonates with the known toxicology of this class. We therefore now frame this saturation as a class-level alert firing combined with a failure to resolve differences within the class, and we use this interpretation to justify the need for the scaffold-aware evaluation presented here.

Application of the descriptor layer to 102 natural compounds of the genus *Heracleum* made it possible to identify a priority set according to several independent, interpretable criteria. Considering the latter, assignment of a compound to a priority tier should be understood as a computational hypothesis with structural support rather than as evidence of a favorable profile. The selection criterion is relative within this natural set rather than absolute. Nevertheless, the transparency of the multi-criteria ranking makes it possible to trace the basis for the selection of each compound and to plan subsequent experimental verification in a targeted manner.

Validation using published experimental data shows that the prioritization scheme selects pharmacologically relevant compounds. Xanthotoxin (methoxsalen) and bergapten are already used in clinical practice as part of PUV-A therapy, meaning that our pipeline (algorithm) independently recovered the known therapeutic agents of this class. The remaining selected compounds are considerably less well studied, yet specific mechanisms of action consistent with therapeutic promise have been described for several of them: sphondin suppresses HBsAg production and cccDNA transcription of hepatitis B virus through degradation of the HBx protein [34]; xanthotoxol exerts neuroprotective and anti-inflammatory effects in focal ischemia and intracerebral hemorrhage [35]; isopimpinellin blocks DNA adduct formation and skin tumor initiation in vivo [36]; pimpinellin inhibits platelet aggregation through the PI3K/Akt pathway [37]; and bergaptol shows anti-inflammatory, antioxidant, and antitumor activity and is the most effective furanocoumarin in suppressing bacterial quorum-sensing [38]. For two further first-tier compounds, the available evidence concerns their closest structural analogs rather than the compounds themselves: heraclesol is the angular counterpart of byakangelicin, which modulates blood–brain barrier permeability, inhibits BACE1, and is neuroprotective in cerebral ischemia [39,40], whereas heraclenol is a positional isomer of oxypeucedanin hydrate, for which a broad spectrum of bioactivities has been reported [41]. Evidence of this kind is weaker than direct experimental data and is treated here only as a prior for experimental follow-up.

Among the compounds of the first priority tier, heraclenol and pabularinone are of the greatest pharmacological interest. Heraclenol has been described as a selective inhibitor of bacterial histidine biosynthesis with antimicrobial and antibiofilm activity against uropathogenic *Escherichia coli* and minimal cytotoxicity toward mammalian cells [42], which corresponds to the combination of selectivity and low systemic toxicity most sought after in an antibacterial candidate. Pabularinone, the only compound in the sample with a favorable profile across all eight informative endpoints simultaneously, exhibits pronounced antiproliferative activity against hepatocellular carcinoma HepG2 with *IC*_50_ = 7.46 µM and against the HeLa line with *IC*_50_ = 13.48 µM [43]. Thus, the prioritization not only reproduces known results but also identifies compounds with documented yet insufficiently investigated therapeutic potential, which makes them rational candidates for priority experimental validation in vitro.

## 4. Materials and Methods

### 4.1. Study Design

**Figure 6:**
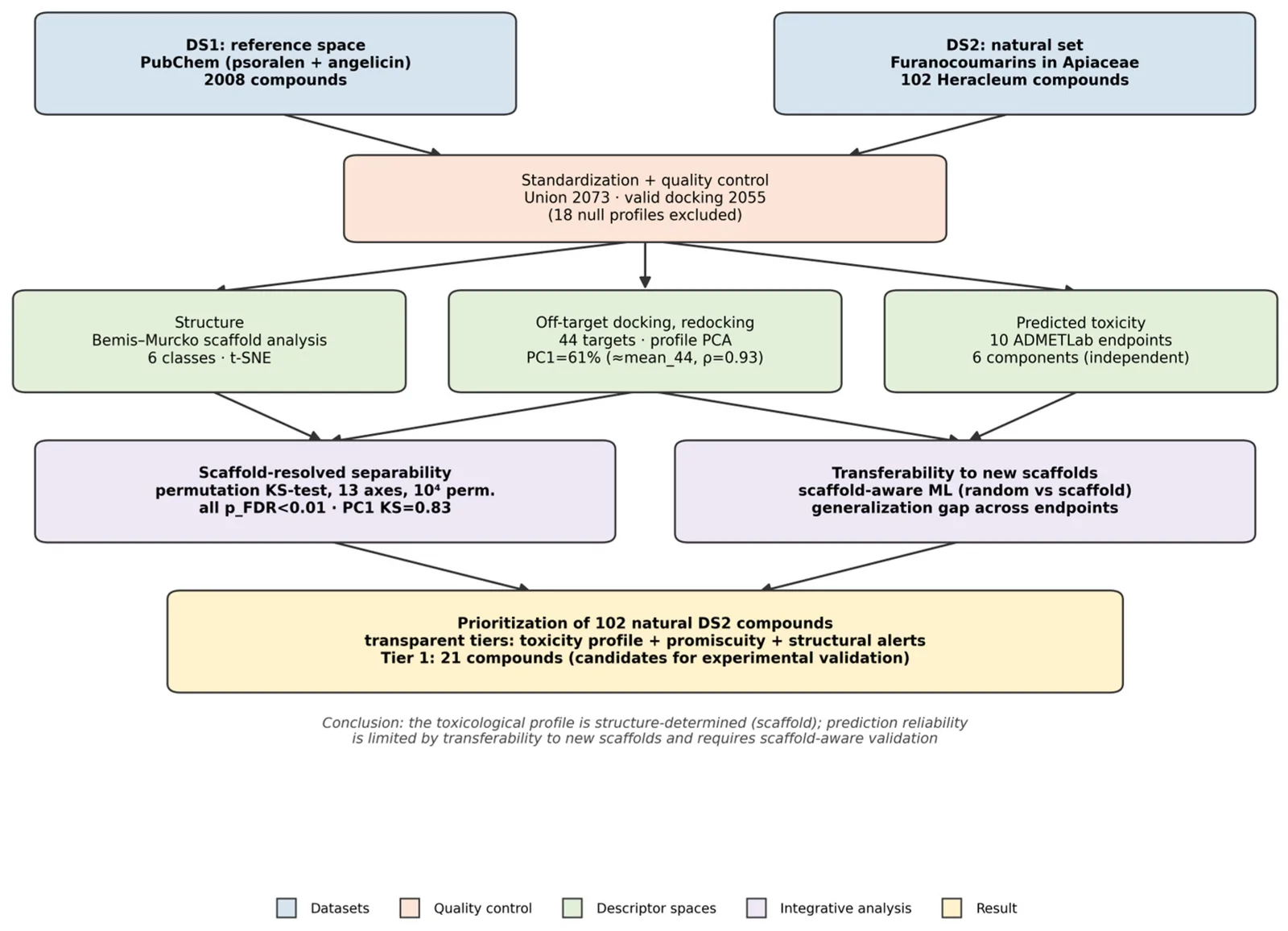
Overall study design and workflow.

Figure 6 depicts the overall study design and workflow.

The pipeline comprised the assembly of two datasets, structure standardization and quality control, construction of three descriptor spaces (structural, off-target binding, and predicted toxicity), assessment of the scaffold-resolved organization of these spaces, analysis of the transferability of structure–property models to new chemical scaffolds, and prioritization of the natural compounds.

### 4.2. Datasets

A dataset of the coumarin and furanocoumarin chemotype comprising 2008 compounds (DS1) was retrieved from the PubChem database [44] using two canonical cores—psoralen and angelicin. A total of 2008 canonical SMILES were obtained, and the compound scaffolds were verified by SMARTS search using the RDKit library (version 2026.3.3) against four substructures covering the linear and angular types in both the aromatic and dihydro forms. Of the 2008 compounds, 2007 were furanocoumarins and 1 was a coumarin (7-methoxy-8-(3-methylbut-2-en-1-yl)-2H-1-benzopyran-2-one). The dataset included both natural furanocoumarins and their synthetic derivatives and was used as a reference space.

The dataset of 145 natural compounds (2327 species–compound records) was obtained from the “Furanocoumarins in *Apiaceae*” database [7,45], which compiles experimentally established coumarins and furanocoumarins of *Heracleum* species of the family *Apiaceae*. In the primary sources the taxa are cited under 97 names, which after resolution of synonymy [46] corresponds to 74 accepted taxa and 60 species. By native range, the species of the sample are distributed as follows [47,48,49]: Europe, the Caucasus and Asia Minor—30 species (60% of the records); East Asia and Siberia—11 species (15%); the Himalayas and South Asia—12 species (11%); Iran and Central Asia—5 species (8%); and North America—1 species (6%), for the remaining species the native range could not be assigned unambiguously. The sample includes both invasive species (*H. mantegazzianum*, *H. sosnowskyi*, *H. persicum*) [49,50] and non-invasive ones, among them species with narrow ranges. Taxon representation is uneven and reflects the history of their study: 65% of the records come from the ten most extensively investigated species. Chemically, the compounds of the sample are divided according to the type of fusion between the furan and coumarin rings: 98 of the 145 structures carry a linear core and 34 an angular one; the remaining 13 are simple coumarins without a fused furan ring.

Angular derivatives were recorded in 52 of the 60 species, and their proportion differs between geographical groups—from 46% of the records in species of Iran, Central Asia and North America to 18.5% in Himalayan and South Asian ones, with the opposite trend for prenylated derivatives. The proportions given reflect the frequency of reports in the literature rather than quantitative content. The dataset contains 145 unique structures. At the time of data collection the database did not provide machine-readable structures, so SMILES strings were retrieved manually for each compound from PubChem on the basis of the reported compound name and CAS number. Canonical SMILES were obtained for 102 compounds, which constitute DS2, while the remaining 43 compounds had no structural match and were excluded from further analysis. After standardization, the union of DS1 and DS2 comprised 2073 unique structures: 1971 in DS1 only, 65 in DS2 only, and 37 present in both sets.

### 4.3. Scaffold Analysis and Chemical Space Analysis

Molecular scaffolds were computed using the Bemis–Murcko method (Bemis–Murcko scaffolds) [51] and visualized using the RDKit library (version 2026.3.3). For each molecule from the standardized SMILES set, a molecular graph was constructed, from which the scaffold—the ring system with linkers but without side substituents—was extracted. In addition, a generic (graph framework) scaffold was computed, in which all atoms are reduced to carbon and all bonds to single bonds, allowing structurally related scaffolds to be grouped irrespective of atom type and bond order. All molecules from DS1 and DS2 underwent SMILES parsing. For the valid set of molecules, the frequency of each unique Bemis–Murcko scaffold and generic scaffold was counted, from which the number of unique scaffolds [52], the mean number of molecules per scaffold, the fraction of singleton scaffolds, and the cumulative coverage—defined as the minimum number of most-represented scaffolds required to describe 50% of the dataset’s molecules—were estimated. The top 15 most frequent scaffolds were ranked by the number of molecules and their fraction of the total valid set. The five most represented scaffolds were assigned the labels Furo 1 (linear psoralen core), Furo 2 (angular angelicin core), Top 1, Top 2, and Top 3; assignment was based on the exact match of the scaffold’s canonical SMILES, so that all molecules within a class share an identical core and within-class variation arises solely from peripheral substituents. The remaining compounds were combined into a heterogeneous Other class, which was excluded from the statistical separability test.

To visualize the 2D chemical space of the DS1 and DS2 datasets, the SynMap module of the *Syntelly* AI platform [53] was used, based on the parametric t-SNE approach [54]. Compounds of the DS2 dataset were projected onto the shared t-SNE map to assess their position within the chemical space of the extended DS1 dataset.

### 4.4. Molecular Docking and Redocking

Molecular docking was performed for 2055 ligands against an off-target panel of 44 proteins, compiled following the secondary-pharmacology panel of Bowes et al. [55] and our earlier antitarget analysis [56], using the scoring function of AutoDock Vina 1.2.5 [57,58] (Supplementary Materials Table S1). In the present study, the docking parameters were set to exhaustiveness = 64 and num_modes = 9. Docking scores were expressed in kcal/mol, with more negative values corresponding to more favorable predicted protein–ligand interactions. For each ligand–target pair, the pose with the best (most negative) docking score was selected. Positive docking-score values were set to 0 prior to the calculation of integrative metrics. For each molecule, the mean docking score (kcal/mol) across all 44 proteins (Mean docking) was calculated.

Negative control compounds were retrieved from the PubChem BioAssay database. Compounds annotated as Inactive, or with a measured activity weaker than 10 µM against the corresponding panel target, were assigned to the negative controls; compounds annotated as Active, or with an activity of 1 µM or better, were assigned to the known actives. Records with non-physical activity values were excluded. Molecular docking of the control compounds was performed using the same protocol as for the main dataset. Differences between the groups were assessed with a one-sided Mann–Whitney test applied separately to each target, with Bonferroni correction for multiple comparisons; the relationship between docking scores and physicochemical properties was characterized by Spearman’s rank correlation coefficient.

#### 4.4.1. Target Preparation

The full-length three-dimensional structures of the 44 biological targets were obtained from the Protein Data Bank [59] and prepared for docking following the protocol described by Nikitin et al. [56].

#### 4.4.2. Ligand Preparation

Salt and counter-ion removal, charge neutralization, tautomer canonicalization, and stereochemistry handling were performed. Following ligand preparation, molecular docking was carried out for 2055 compounds (1953 DS1-only, 37 DS1/DS2 overlap, and 65 DS2-only); 18 molecules (from DS1) were removed from the analysis. After filtering the initial dataset, 3D structures of furanocoumarins in *.pdbqt* format were generated using the Open Babel (*obabel*) [60] package through a series of transformations. First, 2D geometries in *.sdf* format were generated from SMILES with explicit hydrogen atoms added. Then, 3D geometries were generated and optimized using the MMFF94S force field, also in *.sdf* format. These structures were then converted into *.mol2* format with protonation at pH 7.4, and subsequently converted into *.pdbqt* format with calculation of Partial Gasteiger Charges.

#### 4.4.3. Molecular Redocking

The applicability of the present docking setup to the selected antitarget panel was assessed by redocking of co-crystallized ligands, following the approach of Tkachenko et al. [58]. For each crystal structure, the cognate primary ligand was re-docked into its original prepared receptor with AutoDock Vina 1.2.5 using the Vina scoring function, exhaustiveness = 64, num_modes = 20, energy_range = 4 kcal/mol, seed = 42 and a rigid receptor. Symmetry-aware heavy-atom RMSD between the docked and crystallographic poses was computed in the fixed receptor coordinate frame, with atom maps enumerated from the PDBQT REMARK SMILES IDX records and no ligand superposition applied. The reported RMSD is the lowest value among the five top-ranked modes; pose recovery is reported as the first rank at which the RMSD falls within 2.0 Å. Docking scores are reported for the top-ranked pose. Pose recovery within 2.0 Å was assessed cumulatively for the top-ranked pose and for the two, three and five top-ranked modes. Redocking was performed for 42 of the 44 panel targets; the vasopressin V1a receptor (9UWL) and the hERG channel (5VA1) were excluded, the latter being an apo structure with no co-crystallized pharmacological ligand. Per-structure results, including PDB codes, resolutions, grid centers, grid dimensions, ligand codes, docking scores, RMSD values and recovery ranks, are given in Supplementary Materials Table S1a, and the cumulative pose-recovery summary in Table S1b.

### 4.5. Prediction of Toxicological Endpoints

Ten ADMET parameters—respiratory, neurotoxicity, nephrotoxicity, ototoxicity, hematotoxicity, genotoxicity, hepatotoxicity, eye corrosion, eye irritation, and skin sensitization—were computed using ADMETLab 3.0, which is based on D-MPNN models trained on a curated set of more than 400,000 experimental records [23]. The output values are model probabilities rather than binary labels. Calculations were performed using a Python script (version 3.9.6) and the RDKit library (version 2026.3.3) [61]. Two endpoints (genotoxicity, eye irritation), whose distributions are saturated at the upper bound of the scale, were deemed uninformative for ranking and excluded from prioritization.

### 4.6. Scaffold-Aware Machine Learning

The transferability of the structure–property signal to new scaffolds was evaluated for two non-saturated endpoints with high scaffold separability (neurotoxicity, eye corrosion). Elastic Net and Random Forest models were trained to predict the endpoint from Morgan fingerprints under two 5-fold cross-validation schemes: random split and scaffold split (GroupKFold by the generic Bemis–Murcko scaffold), in which all compounds sharing a scaffold are assigned entirely to either the training or the test set. Morgan fingerprints (radius 2, 2048 bits) were computed using a Python script (version 3.9.6) and the RDKit library (version 2026.3.3).

### 4.7. Prioritization of Natural Compounds

The 102 compounds (DS2) were prioritized according to several independent, interpretable criteria. The criteria for a favorable profile were low values across the informative (non-saturated) endpoints—respiratory, neurotoxicity, nephrotoxicity, ototoxicity, hematotoxicity, hepatotoxicity, eye corrosion, and skin sensitization. Genotoxicity and eye irritation were excluded as saturated.

The tiers were defined as follows:

- Tier 1—favorable on ≥6 of 8 endpoints, with low promiscuity (n_strong ≤ 2), Brenk ≤ 1, and no PAINS;
- Tier 2—favorable on ≥5 endpoints, not meeting all Tier 1 criteria, and no PAINS;
- Tier 3—favorable on ≤4 of 8 endpoints.

The structural alerts PAINS [62] and Brenk [63] were computed using the ADMET-AI platform [64]. The tier criteria were fixed prior to inspecting the final list. Compound names were retrieved from PubChem CID identifiers.

### 4.8. Statistical Analysis

All computations were performed in Python (3.9.6), packages Scikit-learn (1.6.1) [65], SciPy (1.13.1) [66], NumPy (2.0.2) [67], Pandas (2.3.3) [68] with a fixed random seed = 42; the significance level was α = 0.05. Principal component analysis (PCA) was applied to z-normalized data separately for the 44 off-target binding profile and the ten toxicity endpoints; the effective dimensionality was determined by the 80% explained-variance threshold. Pairwise relationships among the toxicity endpoints were assessed using the Spearman rank correlation coefficient. The non-randomness of the descriptor-space organization with respect to structure was evaluated along 13 axes (10 toxicity endpoints and the first three principal components of the binding profile, PC1–PC3) for five scaffold classes (Furo 1, Furo 2, Top 1–3; the Other class was excluded). For each axis, the mean pairwise Kolmogorov–Smirnov (KS) distance across all class pairs was computed. Significance was assessed by a permutation test: class labels were randomly permuted 10,000 times while preserving group sizes, and the mean KS was recomputed for each permutation; the *p*-value was defined as the fraction of permutations with a KS no smaller than the observed value. Correction for multiple comparisons across the 13 axes was applied using the Benjamini–Hochberg (FDR) procedure. As a robustness check, the Mann–Whitney U test, Cliff’s delta, and the Hodges–Lehmann shift were additionally computed. The performance of the scaffold-aware models (Section 4.6) was assessed using the coefficient of determination R^2^ on pooled out-of-fold predictions with 95% confidence intervals (bootstrap, 1000 iterations); the random-split and scaffold-split schemes were compared.

## 5. Conclusions

The present work introduces a reproducible computational pipeline that evaluates the consistency and transferability of in silico representations of a chemical class, using the furanocoumarins of the genus *Heracleum* as an example. We show that the chemical space of the class is concentrated around two scaffolds, and that the descriptor space—both docking and toxicological—is non-randomly organized with respect to these scaffolds (all p_FDR < 0.01), meaning that the predicted toxicological profile is determined predominantly by molecular structure. The integrative mean docking score was found to correspond to only the first component of the multidimensional binding profile, while the ten toxicity endpoints are mutually independent and cannot be reduced to a single index. A key result is that the structurally determined signal is only partially transferable to new chemical scaffolds, and the degree of transferability differs between endpoints; consequently, scaffold separability does not guarantee generalizability, and assessment of the generalization gap is required for the correct interpretation of in silico predictions. Based on a transparent multi-criteria prioritization, a candidate set of 21 first-tier compounds was selected from the 102 natural *Heracleum* compounds for subsequent experimental study. The proposed approach separates the consistency of model representations from biological validation and can serve as a basis for the analysis of other taxonomically complex natural classes.

## Data Availability

The data presented in this study are openly available in [GitHub] at [https://github.com/chemagents/heracleum-scaffold-pca] (accessed on 1 August 2026).

## Supplementary Materials

The following supporting information can be downloaded at: https://www.mdpi.com/article/10.3390/ijms27177947/s1.
