# Supplementary material for: From Chemical Scaffold to Predicted Toxicity: A Scaffold-Aware Pipeline for Reliable In Silico Prioritization of Heracleum Furanocoumarins
Source: Int J Mol Sci. 2026 Sep 7;27(17):7947. doi: 10.3390/ijms27177947 (PMC13565919; doi:10.3390/ijms27177947)
Supplement: Supplementary file 1 [file ijms-27-07947-s001.zip › ijms-4497999-supplementary.pdf]

Supplementary Materials

From Chemical Scaffold to Predicted Toxicity: A Scaffold-Aware Pipeline for Reliable In Silico Prioritization of *Heracleum* Furanocoumarins

Anna E. Rassabina[1,2]*, Victor. S. Safronov[1] and Maxim V. Fedorov[1]

[1]Institute for Information Transmission Problems, Russian Academy of Sciences (Kharkevich Institute), Bolshoy Karetny per. 19, Moscow, 127051, Russia; annrassabina@gmail.com (A.E.R.); safronov@phystech.edu (V.S.S.); fedorov@syntelly.com (M.V.F.)
[2]ITMO University, 49 Kronverkskiy Pr., St. Petersburg, 197101, Russia; annrassabina@gmail.com (A.E.R.)
*Correspondence: annrassabina@gmail.com

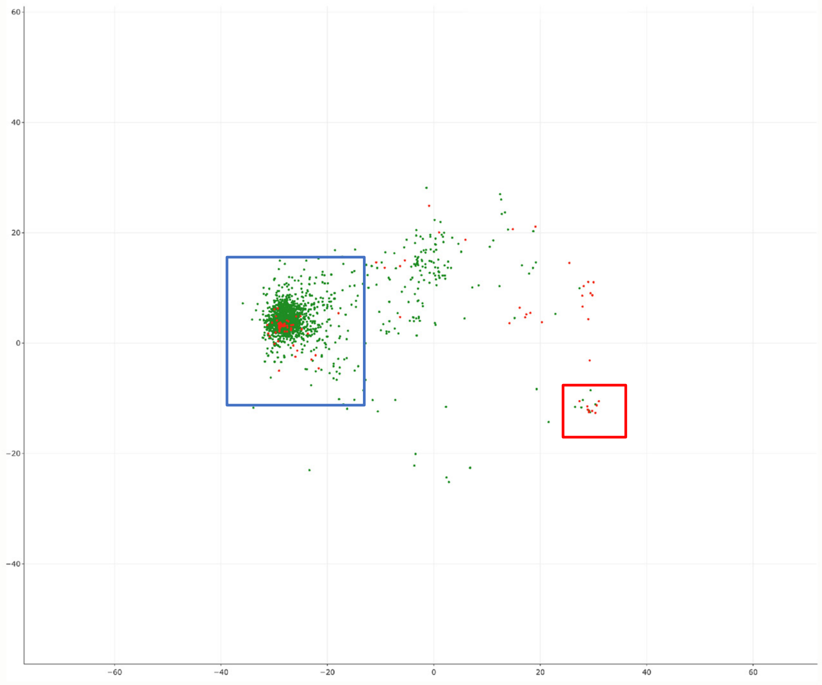

Figure S1. Chemical 2D-space of 2008 compounds (green dots) and 102 compounds of the genus *Heracleum* (red dots), visualized using the SynMap module of the *Syntelly* platform, based on the parametric t-SNE approach. The blue square marks the predominant fraction of furanocoumarin-series compounds, and the red square marks apterin derivatives. In the union of DS1 and DS2, 2073 compounds are observed, 37 compounds are present in both datasets, and 65 are found only in the DS2 dataset.

Table S1a. Target panel of 44 proteins used for docking, with PDB codes, resolutions, grid centres and dimensions, co-crystallized ligand codes, redocking scores, symmetry-aware heavy-atom RMSD values and pose-recovery ranks.

No.	Protein name	Target	PDB	Resolution (Å)	Grid centre (Å)	Grid size (Å)	Ligand	Chemical name	Score (kcal/mol)	RMSD (Å)	Pose recovery	Top-5 status / note
**G protein-coupled receptors**												
1	Adenosine A2A receptor	ADORA2A	2YDO	3.0	-29.06, 3.52, -18.74	17.07, 28.60, 27.81	ADN	Adenosine	-7.591	0.61	rank 1	Recovered in top-5
2	Alpha-1A adrenergic receptor	ADRA1A	7YM8	2.92	138.92, 140.46, 154.54	22.08, 23.32, 29.61	J5C	Oxymetazoline	-8.860	0.56	rank 2	Recovered in top-5
3	Alpha-2A adrenergic receptor	ADRA2A	6KUX	2.7	0.56, -7.80, -23.28	27.82, 25.67, 30.00	E3F	RSC antagonist	-8.471	1.59	rank 1	Recovered in top-5
4	Beta-1 adrenergic receptor	ADRB1	2VT4	2.7	34.73, 14.24, -35.77	25.72, 22.83, 18.34	P32	Cyanopindolol	-8.759	0.53	rank 1	Recovered in top-5
5	Beta-2 adrenergic receptor	ADRB2	2RH1	2.4	-30.12, 4.58, 12.50	27.68, 25.95, 23.03	CAU	(S)-Carazolol	-9.366	0.86	rank 1	Recovered in top-5
6	Cholecystokinin A receptor	CCKAR	7F8Y	2.5	9.01, 24.34, 55.87	24.25, 23.85, 20.64	1OZ	Devazepide	-9.625	1.20	rank 1	Recovered in top-5
7	Cannabinoid receptor 1	CNR1	5U09	2.6	23.35, 1.63, -8.40	18.45, 30.00, 25.08	7DY	Taranabant	-11.572	0.76	rank 3	Recovered in top-5
8	Cannabinoid receptor 2	CNR2	6KPF	2.9	109.34, 107.34, 125.78	25.00, 22.71, 21.74	E3R	E3R	-12.066	0.78	rank 1	Recovered in top-5
9	Dopamine D1 receptor	DRD1	7LJD	3.2	91.57, 108.09, 77.05	26.87, 23.51, 26.75	LDP	Dopamine	-6.172	0.80	rank 3	Recovered in top-5
10	Dopamine D2 receptor	DRD2	6CM4	2.867	11.34, 5.78, -8.67	20.90, 19.48, 27.13	8NU	Risperidone	-12.230	0.39	rank 2	Recovered in top-5
11	Endothelin receptor type A	EDNRA	8XVK	3.21	154.19, 151.54, 169.80	21.94, 26.89, 30.29	A1D5J	Ambrisentan	-7.265	0.83	rank 3	Recovered in top-5
12	Histamine H1 receptor	HRH1	3RZE	3.1	16.61, 33.13, 17.89	24.85, 24.84, 30.75	5EH	E-Doxepin	-11.060	0.79	rank 1	Recovered in top-5
13	Histamine H2 receptor	HRH2	8YN3	2.56	96.25, 112.35, 75.80	16.63, 19.86, 27.00	HSM	Histamine	-4.329	3.25	rank >5	No near-native pose in top-5
14	Delta opioid receptor	OPRD1	4N6H	1.8	-5.44, -72.68, 68.51	19.84, 25.96, 30.00	EJ4	Naltrindole	-11.743	0.27	rank 1	Recovered in top-5
15	Kappa opioid receptor	OPRK1	4DJH	2.9	2.64, -22.05, 64.15	22.48, 23.20, 28.66	JDC	JDTic	-10.860	1.49	rank 2	Recovered in top-5
16	Mu opioid receptor	OPRM1	8EF6	3.2	102.81, 103.58, 133.41	25.28, 26.36, 30.00	MOI	Morphine	-8.300	0.95	rank 1	Recovered in top-5
17	Muscarinic acetylcholine receptor M1	CHRM1	5CXV	2.7	-11.12, -13.34, 37.23	18.28, 18.26, 24.30	0HK	Tiotropium	-9.642	0.50	rank 1	Recovered in top-5
18	Muscarinic acetylcholine receptor M2	CHRM2	4MQS	3.5	-4.52, -11.59, -3.98	17.47, 20.88, 23.16	IXO	Iperoxo	-7.251	1.03	rank 1	Recovered in top-5
19	Muscarinic acetylcholine receptor M3	CHRM3	8E9Y	2.79	124.07, 118.82, 101.19	18.05, 21.99, 17.32	WE9	Clozapine N-oxide (CNO)	-11.340	0.29	rank 1	Recovered in top-5
20	5-hydroxytryptamine receptor 1A	HTR1A	8PJK	2.4	162.74, 171.23, 142.88	19.04, 22.82, 22.42	ZL9	ST171	-8.462	4.43	rank >5	No near-native pose in top-5
21	5-hydroxytryptamine receptor 1B	HTR1B	4IAR	2.7	-11.78, -19.49, 20.00	19.85, 20.57, 24.71	ERM	Ergotamine	-13.740	0.46	rank 1	Recovered in top-5
22	5-hydroxytryptamine receptor 2A	HTR2A	7WC9	2.5	-30.00, -11.88, 143.90	24.06, 25.41, 30.00	93F	Non-hallucinogenic psychedelic analog	-11.040	1.11	rank 4	Recovered in top-5
23	5-hydroxytryptamine receptor 2B	HTR2B	5TVN	2.9	-20.46, -23.56, 11.12	22.04, 32.47, 25.21	7LD	Lysergic acid diethylamide (LSD)	-9.403	0.63	rank 2	Recovered in top-5
24	Vasopressin V1a receptor	AVPR1A	9UWL	N/A	N/A	N/A	N/A	N/A	N/A	N/A	N/A	Native ligand extraction exists, but no prepared PDBQT or completed Vina redocking artifact is available.
**Ion channels**												
25	Neuronal acetylcholine receptor alpha-4 subunit	CHRNA4	8ST0	2.4	219.45, 152.66, 176.16	18.01, 16.17, 17.39	ACH	Acetylcholine	-4.462	0.77	rank 1	Recovered in top-5
26	Voltage-dependent L-type calcium channel alpha-1C subunit	CACNA1C	8FHS	3.3	151.34, 160.77, 147.83	21.97, 29.54, 26.27	WG6	Sofosbuvir	-8.669	7.07	rank >5	No near-native pose in top-5
27	GABA(A) receptor alpha-1 subunit	GABRA1	6X3X	2.92	94.37, 124.75, 104.23	29.14, 18.13, 29.44	DZP	Diazepam	-8.046	0.45	rank 1	Recovered in top-5
28	Potassium voltage-gated channel subfamily H member 2	KCNH2	5VA1	N/A	N/A	N/A	N/A	N/A	N/A	N/A	N/A	Apo structure; no co-crystallized pharmacological ligand is available for pose-recovery redocking.
29	Glutamate receptor ionotropic NMDA 1	GRIN1	1PBQ	1.9	33.10, 27.84, -44.27	30.00, 30.00, 30.00	DK1	5,7-Dichlorokynurenic acid (DCKA)	-8.449	0.91	rank 1	Recovered in top-5
30	5-hydroxytryptamine receptor 3A	HTR3A	6Y1Z	2.82	135.33, 154.63, 159.65	23.22, 18.07, 21.07	O7B	Palonosetron	-11.390	1.94	rank 2	Recovered in top-5
31	Sodium channel protein type 5 alpha subunit	SCN5A	6LQA	3.3	130.16, 125.42, 136.52	27.66, 31.08, 28.79	QDN	Quinidine	-8.443	3.81	rank >5	No near-native pose in top-5
32	Potassium voltage-gated channel subfamily KQT member 1	KCNQ1	7XNK	2.6	134.91, 109.54, 101.76	30.00, 24.34, 24.09	I0S	ML277	-9.218	0.49	rank 2	Recovered in top-5
**Enzymes**												
33	Acetylcholinesterase	ACHE	4EY7	2.3509	-16.96, -44.86, 26.79	30.00, 29.05, 30.00	E20	Donepezil	-12.160	0.32	rank 1	Recovered in top-5

No.	Protein name	Target	PDB	Resolution (Å)	Grid centre (Å)	Grid size (Å)	Ligand	Chemical name	Score (kcal/mol)	RMSD (Å)	Pose recovery	Top-5 status / note
34	Prostaglandin G/H synthase 1	PTGS1	3KK6	2.75	-18.00, 46.50, 40.00	30.00, 27.00, 30.00	CEL	Celecoxib	-9.654	2.74	rank >5	No near-native pose in top-5
35	Prostaglandin G/H synthase 2	PTGS2	3LN1	2.4	32.52, -26.40, -17.98	26.31, 24.54, 25.43	CEL	Celecoxib	-12.592	0.45	rank 1	Recovered in top-5
36	Amine oxidase [flavin-containing] A	MAOA	2Z5X	2.2	39.95, 29.39, -15.40	24.48, 16.12, 17.36	HRM	Harmine	-8.707	4.29	rank >5	No near-native pose in top-5
37	Phosphodiesterase 3A	PDE3A	7KWE	2.0	-18.72, -20.51, 38.75	19.42, 19.99, 30.00	X5M	DNMDP	-8.126	1.74	rank 3	Recovered in top-5
38	Phosphodiesterase 4D	PDE4D	1XOQ	1.83	13.58, 31.59, 57.18	18.68, 22.85, 24.21	ROF	Roflumilast	-9.216	0.83	rank 1	Recovered in top-5
39	Tyrosine-protein kinase Lck	LCK	6PDJ	1.81	-3.54, -0.40, -10.09	30.00, 23.08, 23.99	ODJ	Compound 11	-11.950	1.06	rank 1	Recovered in top-5
**Transporters**												
40	Sodium-dependent noradrenaline transporter	SLC6A2	8WTY	3.2	100.38, 110.56, 110.09	30.00, 29.57, 18.80	XEF	Ziprasidone	-11.160	0.54	rank 2	Recovered in top-5
41	Sodium-dependent dopamine transporter	SLC6A3	9EO4	2.66	130.15, 124.81, 122.84	21.98, 20.57, 27.81	COC	Cocaine	-8.995	0.91	rank 1	Recovered in top-5
42	Sodium-dependent serotonin transporter	SLC6A4	5I71	3.15	-38.12, -16.46, 3.04	30.00, 24.43, 20.20	68P	Escitalopram	-9.668	0.93	rank 1	Recovered in top-5
**Nuclear receptors**												
43	Androgen receptor	AR	3B66	1.65	27.23, 2.37, 6.63	18.10, 19.47, 23.11	B66	SARM S-21	-11.000	0.93	rank 1	Recovered in top-5
44	Glucocorticoid receptor	NR3C1	1M2Z	2.5	19.11, 26.70, -10.88	17.51, 24.05, 24.32	DEX	Dexamethasone	-12.760	0.39	rank 1	Recovered in top-5

Table S1b. Cumulative pose-recovery summary of the redocking experiments (fixed-frame RMSD ≤ 2 Å).

Rank window	Ligands recovered	Rate
Top-1	24/42	57.1%
Top-2	31/42	73.8%
Top-3	35/42	83.3%
Top-5	36/42	85.7%

Table S1c. Panel targets with the corresponding molecular initiating events (MIE) and potential adverse outcomes.

No	Target	PDB code	Molecular initiating event (MIE)	Potential adverse outcome
1	ADORA2A	2YDO	A2A receptor modulation	CNS and cardiovascular effects
2	ADRA1A	7YM8	Alpha-1A modulation	Orthostatic hypotension
3	ADRA2A	6KUX	Alpha-2A modulation	Sedation, hypotension
4	ADRB1	2VT4	Beta-1 modulation	Altered heart rate and contractility
5	ADRB2	2RH1	Beta-2 modulation	Bronchoconstriction, tremor
6	AVPR1A	9UWL	V1A modulation	Vasoconstriction, water balance
7	CCKAR	7F8Y	CCK-A modulation	Gastrointestinal effects
8	CHRM1	5CXV	M1 antagonism	Cognitive impairment
9	CHRM2	4MQS	M2 antagonism	Tachycardia
10	CHRM3	8E9Y	M3 antagonism	Dry mouth, urinary retention, GI effects
11	CNR1	5U09	CB1 modulation	CNS effects, appetite changes
12	CNR2	6KPF	CB2 modulation	Immune modulation
13	DRD1	7LJD	D1 modulation	Motor and reward effects
14	DRD2	6CM4	D2 antagonism	Extrapyramidal symptoms, hyperprolactinaemia
15	EDNRA	8XVK	ET-A modulation	Vasoconstriction, hypertension
16	HRH1	3RZE	H1 antagonism	Sedation, weight gain
17	HRH2	8YN3	H2 antagonism	Gastric acid suppression

No	Target	PDB code	Molecular initiating event (MIE)	Potential adverse outcome
18	HTR1A	8PJK	5-HT1A modulation	Anxiety, thermoregulatory effects
19	HTR1B	4IAR	5-HT1B modulation	Vasoconstriction, CNS effects
20	HTR2A	7WC9	5-HT2A modulation	Hallucinations, CNS effects
21	HTR2B	5TVN	5-HT2B agonism	Cardiac valvulopathy
22	OPRD1	4N6H	Delta-opioid modulation	CNS effects, seizure risk
23	OPRK1	4DJH	Kappa-opioid modulation	Dysphoria, sedation
24	OPRM1	8EF6	Mu-opioid modulation	Respiratory depression, dependence
25	CHRNA4	8ST0	Nicotinic alpha-4 modulation	Seizure risk, cognitive effects
26	GABRA1	6X3X	GABA-A modulation	Sedation, dependence, seizure risk
27	GRIN1	1PBQ	NMDA receptor modulation	Excitotoxicity, psychotomimetic effects
28	HTR3A	6Y1Z	5-HT3 modulation	Emesis, altered GI motility
29	CACNA1C	8FHS	Cav1.2 block	Negative inotropy, hypotension
30	KCNH2	5VA1	hERG block	QT prolongation, torsade de pointes
31	KCNQ1	7XNK	IKs block	QT prolongation
32	SCN5A	6LQA	Nav1.5 block	Conduction slowing, arrhythmia
33	AR	3B66	Androgen receptor modulation	Endocrine disruption, reproductive toxicity
34	NR3C1	1M2Z	Glucocorticoid receptor modulation	Immunosuppression, metabolic effects
35	SLC6A2	8WTY	NET inhibition	Cardiovascular and CNS effects
36	SLC6A3	9EO4	DAT inhibition	Abuse liability, CNS stimulation
37	SLC6A4	5I71	SERT inhibition	Serotonin syndrome, CNS effects
38	ACHE	4EY7	AChE inhibition	Cholinergic crisis, neurotoxicity
39	MAOA	2Z5X	MAO-A inhibition	Hypertensive crisis, serotonin syndrome
40	PDE3A	7KWE	PDE3 inhibition	Positive inotropy, arrhythmia
41	PDE4D	1XOQ	PDE4 inhibition	Emesis, nausea
42	PTGS1	3KK6	COX-1 inhibition	Gastrointestinal ulceration, bleeding
43	PTGS2	3LN1	COX-2 inhibition	Cardiovascular thrombotic events
44	LCK	6PDJ	Lck inhibition	Immunosuppression

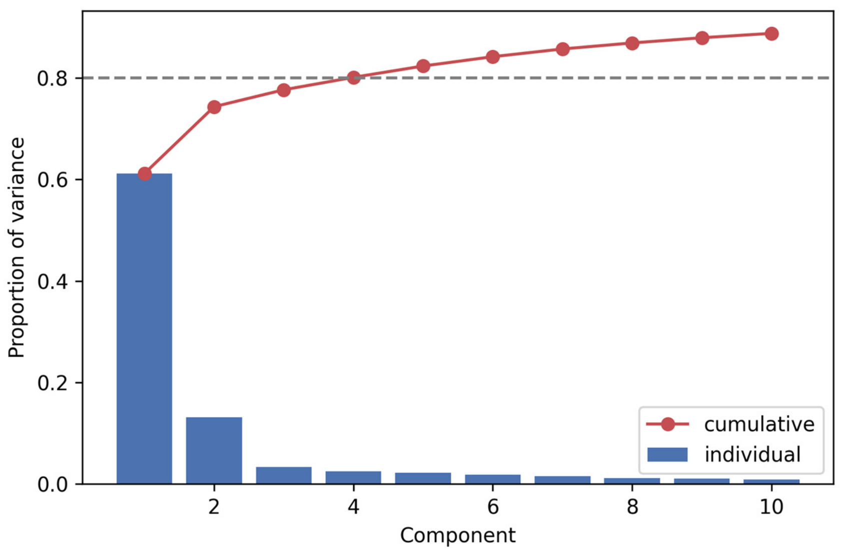

Figure S2. Scree plot of the off-target binding profile PCA with the 80% explained-variance cut-off.

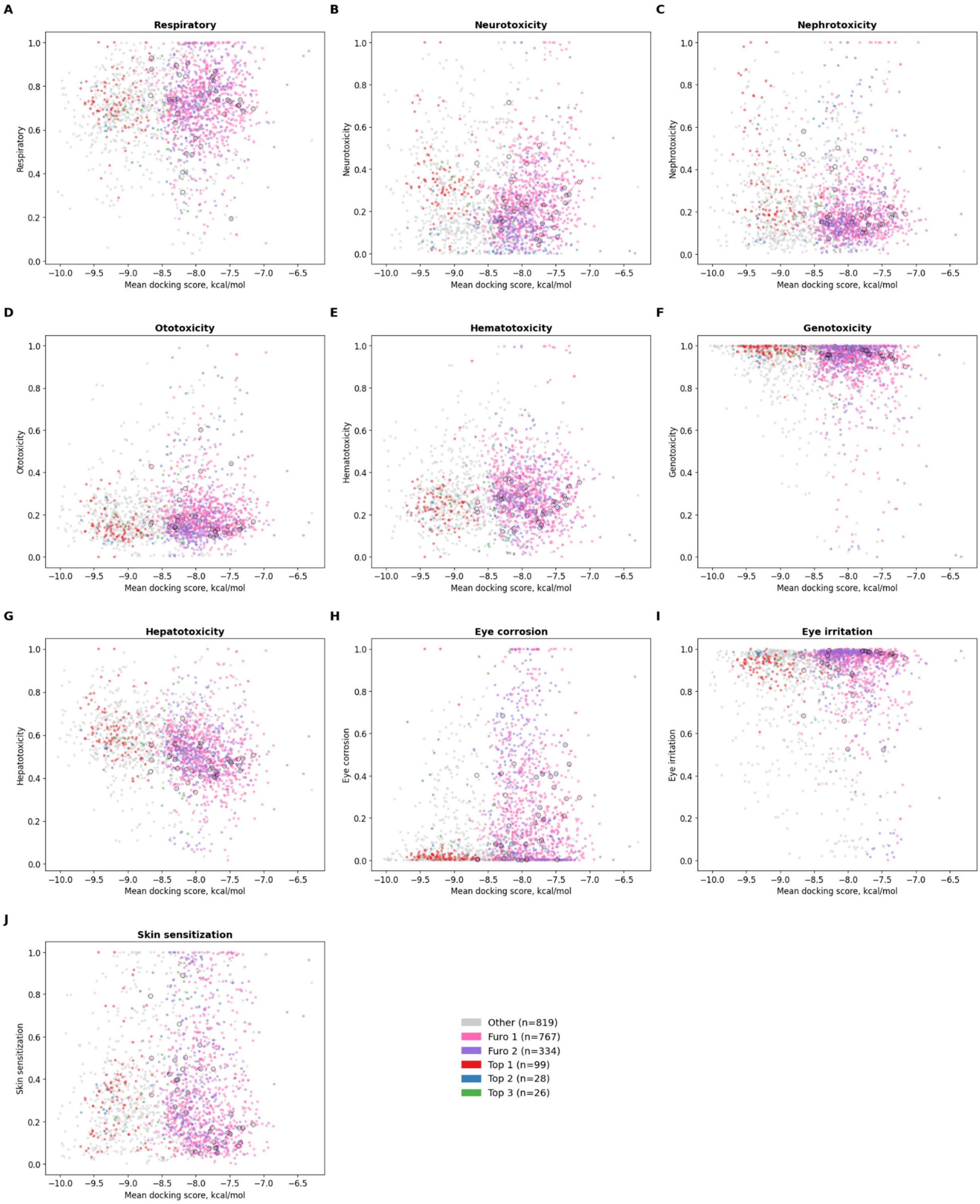

Figure S3. 10 scatter-plot panels: the X-axis shows the mean docking score (44 targets) in kcal/mol, and the Y-axis shows the corresponding toxicity endpoint value: (A) Respiratory, (B) Neurotoxicity, (C) Nephrotoxicity, (D) Ototoxicity, (E) Hematotoxicity, (F) Genotoxicity, (G) Hepatotoxicity, (H) Eye corrosion, (I) Eye irritation, and (J) Skin sensitization. Points are colored according to the compound's scaffold group membership: gray — Other (n=819), pink — Furo 1 (n=767), purple — Furo 2 (n=334), red — Top 1 (n=99), blue — Top 2 (n=28), green — Top 3 (n=26). A black outline additionally highlights the 102 compounds from the genus *Heracleum* containing a furan-containing fragment, regardless of their group membership.

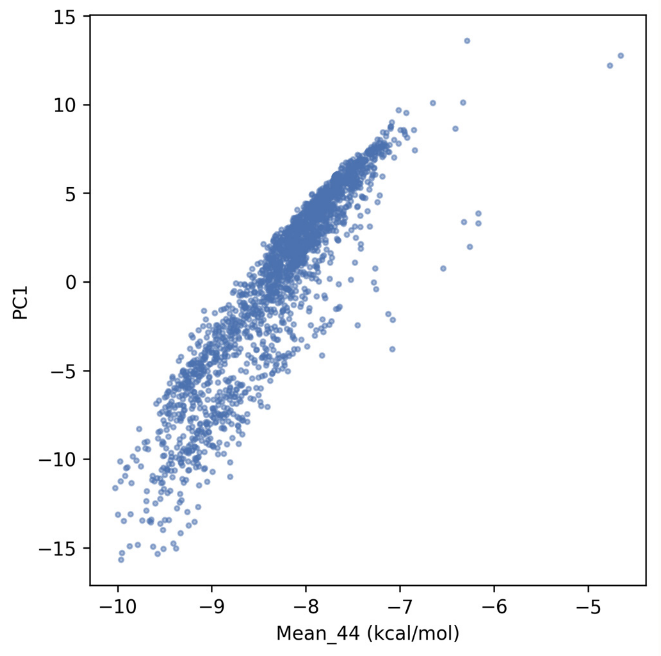

Figure S4. Relationship between the first principal component of the binding profile and the mean docking score across the 44 targets (ρ = 0.927).

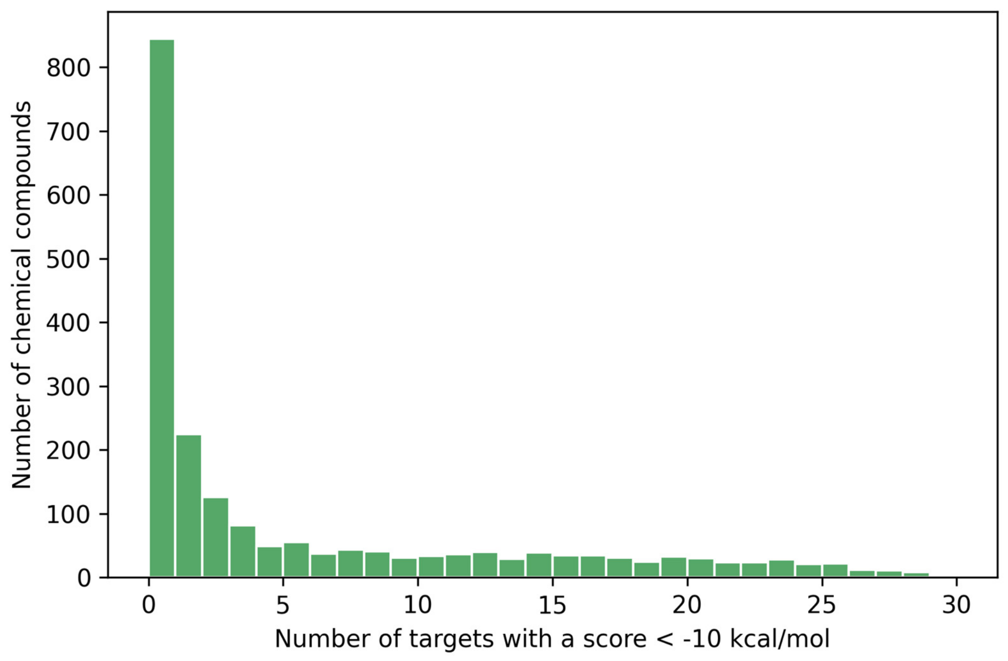

Figure S5. Distribution of promiscuity (number of strong targets) across scaffold classes.

Table S2. Descriptive statistics of the ten predicted toxicity endpoints (median, share of values > 0.9 and < 0.1, interquartile range). The values that are discussed in the main text of the article are highlighted in bold.

**Endpoint**	**Median**	**Share > 0.9**	**Share < 0.1**	**IQR**
Respiratory	0.715	0.116	0.006	**0.194**
Neurotoxicity	0.231	0.023	0.182	**0.252**
Nephrotoxicity	0.185	0.019	0.190	0.197
Ototoxicity	0.175	0.005	0.131	0.126
Hematotoxicity	0.282	0.008	0.056	0.164
**Genotoxicity**	**0.958**	**0.748**	0.011	0.089
Hepatotoxicity	0.547	0.017	0.009	0.173
Eye corrosion	0.078	0.026	0.540	**0.280**
**Eye irritation**	**0.945**	**0.655**	0.027	0.140
Skin sensitization	0.286	0.077	0.134	**0.342**

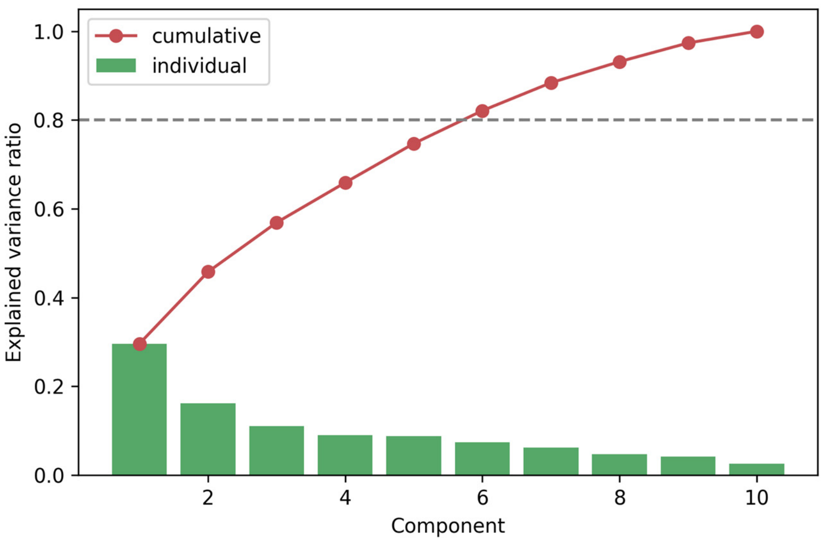

Figure S6. ADMET PCA, 6 components needed for 80%.

Table S3a. Point estimates of R^2, RMSE, MAE and RMSE/SD for Random Forest and Elastic Net under random-split and scaffold-split five-fold cross-validation.

Endpoint	Model	Split	R^2	RMSE	MAE	RMSE/SD
Neurotoxicity	RF	random	0.645	0.127	0.086	0.60
Neurotoxicity	RF	scaffold	0.444	0.158	0.113	0.75
Neurotoxicity	Elastic Net	random	0.310	0.177	0.133	0.83
Neurotoxicity	Elastic Net	scaffold	0.195	0.191	0.146	0.90
Eye corrosion	RF	random	0.670	0.142	0.091	0.57
Eye corrosion	RF	scaffold	0.308	0.205	0.137	0.83
Eye corrosion	Elastic Net	random	0.357	0.198	0.145	0.80
Eye corrosion	Elastic Net	scaffold	0.162	0.226	0.161	0.92

Table S3b. Percentile bootstrap 95% confidence intervals (1000 resamples).

Endpoint	Model	Split	R^2 lower	R^2 upper	RMSE lower	RMSE upper	MAE lower	MAE upper
Neurotoxicity	RF	random	0.606	0.680	0.120	0.134	0.082	0.090
Neurotoxicity	RF	scaffold	0.397	0.490	0.150	0.166	0.108	0.118
Neurotoxicity	Elastic Net	random	0.283	0.337	0.169	0.184	0.128	0.138
Neurotoxicity	Elastic Net	scaffold	0.167	0.223	0.183	0.198	0.141	0.152
Eye corrosion	RF	random	0.623	0.710	0.133	0.151	0.086	0.096
Eye corrosion	RF	scaffold	0.251	0.358	0.195	0.215	0.131	0.144
Eye corrosion	Elastic Net	random	0.330	0.382	0.189	0.207	0.139	0.150
Eye corrosion	Elastic Net	scaffold	0.137	0.186	0.216	0.236	0.155	0.168

Table S4. Natural *Heracleum* compounds assigned to the first priority tier (Tier 1): favorable predicted profile across ≥6 of 8 informative toxicity endpoints, low off-target promiscuity, and absence of structural alerts (PAINS = 0, Brenk ≤ 1).

№	InChIKey	SMILES	Name	IUPAC name
1	ZRSSNKQAHFVTPW-UHFFFAOYSA-N	COc1coc2cc3oc(=O)ccc3cc12	—	3-methoxyfuro[3,2-g]chromen-7-one
2	MVJHUMZXIJPVHV-UHFFFAOYSA-N	COc1c2ccoc2c(O)c2oc(=O)ccc12	—	8-Hydroxybergapten
3	XDROKJSWHURZGO-UHFFFAOYSA-N	O=c1ccc2ccc3occc3c2o1	Angelicin	2H-furo[2,3-h][1]benzopyran-2-one
4	ZKCVFMVZXPEHMD-UHFFFAOYSA-N	O=c1ccc2cc(O)c3occc3c2o1	Heratomol	6-hydroxyfuro[2,3-h]chromen-2-one
5	QXKHYNVANLEOEG-UHFFFAOYSA-N	COc1c2occc2cc2ccc(=O)oc12	Xanthotoxin	9-methoxy-7H-furo[3,2-g][1]benzopyran-7-one
6	BGEBZHIAGXMEMV-UHFFFAOYSA-N	COc1c2ccoc2cc2oc(=O)ccc12	Bergapten	4-methoxy-7H-furo[3,2-g][1]benzopyran-7-one
7	JWVYQQGERKEAHW-UHFFFAOYSA-N	O=c1ccc2cc3ccoc3c(O)c2o1	Xanthotoxol	—
8	AJSPSRWWZBBIOR-UHFFFAOYSA-N	COc1cc2occc2c2oc(=O)ccc12	Isobergaptene	—
9	KDJVHSVOXOZBDR-UHFFFAOYSA-N	CC(C)(O)c1cc2c(ccc3ccc(=O)oc32)o1	Oroselol	8-(2-hydroxypropan-2-yl)furo[2,3-h]chromen-2-one
10	GIJHDGJRTUSBJR-UHFFFAOYSA-N	O=c1ccc2c(O)c3ccoc3cc2o1	Bergaptol	—
11	BQPRWZCEKZLBHL-UHFFFAOYSA-N	COc1c(OC)c2occc2c2oc(=O)ccc12	Pimpinellin	—
12	DFMAXQKDIGCMTL-UHFFFAOYSA-N	COc1c2ccoc2c(OC)c2oc(=O)ccc12	Isopimpinellin	4,9-dimethoxy-7H-furo[3,2-g][1]benzopyran-7-one

№	InChIKey	SMILES	Name	IUPAC name
13	SYCQEMIWUFVJHN-LLVKDONJSA-N	COc1c(OC[C@@H](O)C(C)(C)O)c2occc2c2oc(=O)ccc12	Heraclesol	6-[(2R)-2,3-dihydroxy-3-methylbutoxy]-5-methoxyfuro[2,3-h]chromen-2-one
14	ZCCUUQDIBDJBTK-UHFFFAOYSA-N	O=c1ccc2cc3ccoc3cc2o1	Psoralen	—
15	USLPJJIUMAKBIU-UHFFFAOYSA-N	CC(C)C(=O)COc1c2ccoc2cc2oc(=O)ccc12	Pabulenone	4-(3-methyl-2-oxobutoxy)furo[3,2-g]chromen-7-one
16	DLCJNIBLOSKIQW-UHFFFAOYSA-N	COc1cc2ccc(=O)oc2c2ccoc12	Sphondin	6-methoxy-2H-furo[2,3-h][1]benzopyran-2-one
17	SVPDNNKLQWHTPC-UHFFFAOYSA-N	CC(C)C(=O)COc1c2occc2cc2ccc(=O)oc12	Pabularinone	9-(3-methyl-2-oxobutoxy)furo[3,2-g]chromen-7-one
18	OOWBIFOFDYBTAE-UHFFFAOYSA-N	O=c1ccc2c(O)cc3occc3c2o1	Isobergaptol	5-hydroxyfuro[2,3-h]chromen-2-one
19	FOINLJRVEBYARJ-LLVKDONJSA-N	CC(C)(O)[C@H](O)COc1c2occc2cc2ccc(=O)oc12	Heraclenol	—
20	IQTTZQQJJBEAIM-ZDUSSCGKSA-N	CC(=O)OC(C)(C)[C@@H]1Cc2c(ccc3ccc(=O)oc23)O1	(+)-Columbianetin acetate	—
21	FDGAHDVLSOQOKB-XKKJXBDVSA-N	CC(C)(O)[C@H](COc1c2occc2cc2ccc(=O)oc12)OC(C)(C)[C@@H](COc1c2occc2cc2ccc(=O)oc12)OC(C)(C)[C@H](O)COc1c2occc2cc2ccc(=O)oc12	—	—

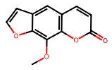

COc1c2occc2cc2ccc(=O)oc12

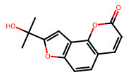

CC(C)(O)c1cc2c(ccc3ccc(=O)oc32)o1

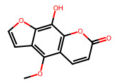

COc1c2ccoc2c(O)c2oc(=O)ccc12

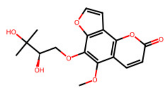

COc1c(OC[C@@H](O)C(C)(C)O)c2occc2c2oc(=O)ccc12

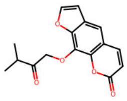

CC(C)C(=O)COc1c2occc2cc2ccc(=O)oc12

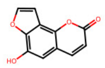

O=c1ccc2cc(O)c3occc3c2o1

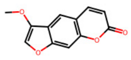

COc1coc2cc3oc(=O)ccc3cc12

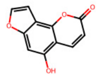

O=c1ccc2c(O)cc3occc3c2o1

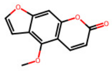

COc1c2ccoc2cc2oc(=O)ccc12

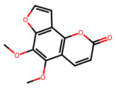

COc1c(OC)c2occc2c2oc(=O)ccc12

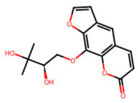

CC(C)(O)[C@H](O)COc1c2occc2cc2ccc(=O)oc12

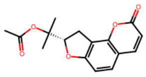

CC(=O)OC(C)(C)[C@@H]1Cc2c(ccc3ccc(=O)oc23)O1

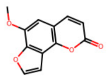

COc1cc2ccc(=O)oc2c2ccoc12

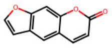

O=c1ccc2cc3ccoc3cc2o1

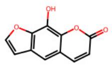

O=c1ccc2cc3ccoc3c(O)c2o1

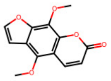

COc1c2ccoc2c(OC)c2oc(=O)ccc12

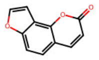

O=c1ccc2ccc3occc3c2o1

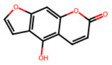

O=c1ccc2c(O)c3ccoc3cc2o1

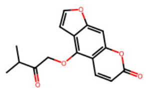

CC(C)C(=O)COc1c2ccoc2cc2oc(=O)ccc12

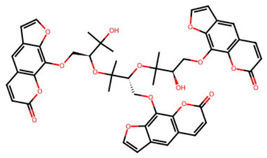

CC(C)(O)[C@H](COc1c2occc2cc2ccc(=O)oc12)OC(C)(C)[C@@H](COc1c2occc2cc2ccc(=O)oc12)OC(C)(C)[C@H](O)COc1c2occc2cc2ccc(=O)oc12

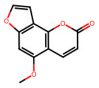

COc1cc2occc2c2oc(=O)ccc12

Figure S7. 2D structures of all 21 compounds.

Table S5. Scaffold-level separability of the descriptor axes: observed and random-label mean pairwise Kolmogorov–Smirnov distances between the five scaffold classes, with permutation-based 95% intervals and p-values. The mean KS distance is the average pairwise Kolmogorov–Smirnov distance across the Furo 1, Furo 2, Top 1, Top 2 and Top 3 classes; random-label values were obtained from 10,000 permutations preserving group sizes. Class sizes refer to the compounds with a valid 44-dimensional docking profile and are therefore slightly smaller than the scaffold counts in Figure 1A.

Scaffold-level separability across toxicity/docking descriptor axes

Axis	N	Observed mean KS distance	Random-label mean KS distance	Random-label 95% interval	Permutation p	Use for follow-up
Mean docking	1245	0.599	0.148	0.107-0.203	<0.001	binding-axis priority
Neurotoxicity	1245	0.509	0.149	0.105-0.211	<0.001	toxicity follow-up priority
Eye corrosion	1245	0.445	0.149	0.104-0.208	<0.001	irritation/corrosion follow-up
Nephrotoxicity	1245	0.379	0.150	0.106-0.213	<0.001	nephrotoxicity follow-up
Ototoxicity	1245	0.350	0.148	0.107-0.204	<0.001	ototoxicity follow-up
Respiratory	1245	0.304	0.151	0.108-0.206	<0.001	respiratory signal
Hematotoxicity	1245	0.304	0.148	0.104-0.206	<0.001	hematotoxicity signal
Eye irritation	1245	0.300	0.146	0.106-0.200	<0.001	lower-priority signal
Hepatotoxicity	1245	0.296	0.148	0.106-0.205	<0.001	lower-priority signal
Genotoxicity	1245	0.286	0.150	0.106-0.210	<0.001	lower-priority signal
Skin sensitization	1245	0.274	0.147	0.103-0.206	<0.001	weakest axis

Table S6a. Pairwise robustness analysis for all class pairs along each descriptor axis, giving group sizes, Mann–Whitney q-values, Cliff's delta and the Hodges–Lehmann shift. Part 1.

Supplementary pairwise robustness table, part 1/4

Axis	Pair	nA/nB	MWU q	Cliff's delta	HL shift	Strong
Mean docking	Furo 1 vs Furo 2	760/333	0.021	0.095	0.059	no
Mean docking	Furo 1 vs Top 1	760/98	<0.001	0.992	1.257	yes
Mean docking	Furo 1 vs Top 2	760/28	<0.001	0.689	0.689	yes
Mean docking	Furo 1 vs Top 3	760/26	<0.001	0.779	0.570	yes
Mean docking	Furo 2 vs Top 1	333/98	<0.001	0.992	1.197	yes
Mean docking	Furo 2 vs Top 2	333/28	<0.001	0.653	0.620	yes
Mean docking	Furo 2 vs Top 3	333/26	<0.001	0.755	0.498	no
Mean docking	Top 1 vs Top 2	98/28	<0.001	-0.477	-0.597	yes
Mean docking	Top 1 vs Top 3	98/26	<0.001	-0.794	-0.707	yes
Mean docking	Top 2 vs Top 3	28/26	0.511	-0.122	-0.099	no
Neurotoxicity	Furo 1 vs Furo 2	760/333	<0.001	0.308	0.075	no
Neurotoxicity	Furo 1 vs Top 1	760/98	<0.001	-0.270	-0.071	no
Neurotoxicity	Furo 1 vs Top 2	760/28	<0.001	0.866	0.205	yes
Neurotoxicity	Furo 1 vs Top 3	760/26	0.088	0.224	0.050	no
Neurotoxicity	Furo 2 vs Top 1	333/98	<0.001	-0.514	-0.150	yes
Neurotoxicity	Furo 2 vs Top 2	333/28	<0.001	0.733	0.122	yes
Neurotoxicity	Furo 2 vs Top 3	333/26	0.361	-0.124	-0.027	no
Neurotoxicity	Top 1 vs Top 2	98/28	<0.001	0.913	0.284	yes
Neurotoxicity	Top 1 vs Top 3	98/26	0.001	0.448	0.125	no
Neurotoxicity	Top 2 vs Top 3	28/26	<0.001	-0.843	-0.145	yes
Eye corrosion	Furo 1 vs Furo 2	760/333	0.005	-0.114	-0.040	no
Eye corrosion	Furo 1 vs Top 1	760/98	<0.001	0.672	0.146	yes
Eye corrosion	Furo 1 vs Top 2	760/28	0.233	0.151	0.039	no
Eye corrosion	Furo 1 vs Top 3	760/26	0.279	0.140	0.033	no
Eye corrosion	Furo 2 vs Top 1	333/98	<0.001	0.623	0.188	yes
Eye corrosion	Furo 2 vs Top 2	333/28	0.102	0.213	0.075	no
Eye corrosion	Furo 2 vs Top 3	333/26	0.104	0.218	0.082	no
Eye corrosion	Top 1 vs Top 2	98/28	<0.001	-0.728	-0.057	no

Cliff's delta and HL shift are signed for group A minus group B. Strong = q_KS<0.001, q_MWU<0.001, KS D>=0.30 and axis-specific HL-shift threshold met.

Table S6b. Pairwise robustness analysis for all class pairs along each descriptor axis, giving group sizes, Mann–Whitney q-values, Cliff's delta and the Hodges–Lehmann shift. Part 2.

Supplementary pairwise robustness table, part 2/4

Axis	Pair	nA/nB	MWU q	Cliff's delta	HL shift	Strong
Eye corrosion	Top 1 vs Top 3	98/26	<0.001	-0.631	-0.086	no
Eye corrosion	Top 2 vs Top 3	28/26	0.783	-0.048	-0.008	no
Nephrotoxicity	Furo 1 vs Furo 2	760/333	0.152	0.061	0.010	no
Nephrotoxicity	Furo 1 vs Top 1	760/98	<0.001	-0.473	-0.099	no
Nephrotoxicity	Furo 1 vs Top 2	760/28	<0.001	-0.408	-0.328	yes
Nephrotoxicity	Furo 1 vs Top 3	760/26	0.514	-0.086	-0.020	no
Nephrotoxicity	Furo 2 vs Top 1	333/98	<0.001	-0.502	-0.108	yes
Nephrotoxicity	Furo 2 vs Top 2	333/28	<0.001	-0.434	-0.324	yes
Nephrotoxicity	Furo 2 vs Top 3	333/26	0.376	-0.119	-0.029	no
Nephrotoxicity	Top 1 vs Top 2	98/28	0.262	-0.158	-0.102	no
Nephrotoxicity	Top 1 vs Top 3	98/26	0.011	0.353	0.107	no
Nephrotoxicity	Top 2 vs Top 3	28/26	0.005	0.484	0.320	no
Ototoxicity	Furo 1 vs Furo 2	760/333	<0.001	0.216	0.030	no
Ototoxicity	Furo 1 vs Top 1	760/98	<0.001	0.385	0.044	no
Ototoxicity	Furo 1 vs Top 2	760/28	<0.001	-0.408	-0.183	yes
Ototoxicity	Furo 1 vs Top 3	760/26	0.001	0.395	0.051	no
Ototoxicity	Furo 2 vs Top 1	333/98	0.104	0.123	0.014	no
Ototoxicity	Furo 2 vs Top 2	333/28	<0.001	-0.465	-0.196	yes
Ototoxicity	Furo 2 vs Top 3	333/26	0.186	0.176	0.021	no
Ototoxicity	Top 1 vs Top 2	98/28	<0.001	-0.580	-0.253	yes
Ototoxicity	Top 1 vs Top 3	98/26	0.668	0.063	0.006	no
Ototoxicity	Top 2 vs Top 3	28/26	<0.001	0.578	0.246	yes
Respiratory	Furo 1 vs Furo 2	760/333	0.540	0.026	0.006	no
Respiratory	Furo 1 vs Top 1	760/98	0.507	0.048	0.010	no
Respiratory	Furo 1 vs Top 2	760/28	<0.001	0.430	0.101	yes
Respiratory	Furo 1 vs Top 3	760/26	<0.001	0.450	0.130	yes
Respiratory	Furo 2 vs Top 1	333/98	0.734	0.025	0.005	no
Respiratory	Furo 2 vs Top 2	333/28	0.002	0.381	0.096	no

Cliff's delta and HL shift are signed for group A minus group B. Strong = q_KS<0.001, q_MWU<0.001, KS D>=0.30 and axis-specific HL-shift threshold met.

Table S6c. Pairwise robustness analysis for all class pairs along each descriptor axis, giving group sizes, Mann–Whitney q-values, Cliff's delta and the Hodges–Lehmann shift. Part 3.

Supplementary pairwise robustness table, part 3/4

Axis	Pair	nA/nB	MWU q	Cliff's delta	HL shift	Strong
Respiratory	Furo 2 vs Top 3	333/26	<0.001	0.420	0.122	yes
Respiratory	Top 1 vs Top 2	98/28	<0.001	0.450	0.088	no
Respiratory	Top 1 vs Top 3	98/26	<0.001	0.468	0.116	no
Respiratory	Top 2 vs Top 3	28/26	0.684	0.074	0.020	no
Hematotoxicity	Furo 1 vs Furo 2	760/333	0.109	0.069	0.014	no
Hematotoxicity	Furo 1 vs Top 1	760/98	<0.001	0.282	0.052	no
Hematotoxicity	Furo 1 vs Top 2	760/28	0.383	-0.111	-0.027	no
Hematotoxicity	Furo 1 vs Top 3	760/26	<0.001	0.433	0.108	yes
Hematotoxicity	Furo 2 vs Top 1	333/98	0.004	0.208	0.037	no
Hematotoxicity	Furo 2 vs Top 2	333/28	0.210	-0.161	-0.042	no
Hematotoxicity	Furo 2 vs Top 3	333/26	0.002	0.383	0.093	no
Hematotoxicity	Top 1 vs Top 2	98/28	0.012	-0.339	-0.081	no
Hematotoxicity	Top 1 vs Top 3	98/26	0.140	0.214	0.056	no
Hematotoxicity	Top 2 vs Top 3	28/26	0.003	0.505	0.132	no
Eye irritation	Furo 1 vs Furo 2	760/333	0.146	-0.062	-0.003	no
Eye irritation	Furo 1 vs Top 1	760/98	<0.001	0.300	0.028	no
Eye irritation	Furo 1 vs Top 2	760/28	0.190	0.164	0.013	no
Eye irritation	Furo 1 vs Top 3	760/26	0.243	0.152	0.012	no
Eye irritation	Furo 2 vs Top 1	333/98	<0.001	0.337	0.032	no
Eye irritation	Furo 2 vs Top 2	333/28	0.105	0.209	0.014	no
Eye irritation	Furo 2 vs Top 3	333/26	0.135	0.201	0.015	no
Eye irritation	Top 1 vs Top 2	98/28	0.734	0.047	0.009	no
Eye irritation	Top 1 vs Top 3	98/26	0.146	-0.208	-0.017	no
Eye irritation	Top 2 vs Top 3	28/26	0.530	-0.115	-0.018	no
Hepatotoxicity	Furo 1 vs Furo 2	760/333	0.975	0.001	0.000	no
Hepatotoxicity	Furo 1 vs Top 1	760/98	<0.001	-0.460	-0.096	no
Hepatotoxicity	Furo 1 vs Top 2	760/28	0.044	-0.248	-0.041	no
Hepatotoxicity	Furo 1 vs Top 3	760/26	0.805	-0.030	-0.006	no

Cliff's delta and HL shift are signed for group A minus group B. Strong = q_KS<0.001, q_MWU<0.001, KS D>=0.30 and axis-specific HL-shift threshold met.

Table S6d. Pairwise robustness analysis for all class pairs along each descriptor axis, giving group sizes, Mann–Whitney q-values, Cliff's delta and the Hodges–Lehmann shift. Part 4.

Supplementary pairwise robustness table, part 4/4

Axis	Pair	nA/nB	MWU q	Cliff's delta	HL shift	Strong
Hepatotoxicity	Furo 2 vs Top 1	333/98	<0.001	-0.483	-0.095	no
Hepatotoxicity	Furo 2 vs Top 2	333/28	0.021	-0.286	-0.043	no
Hepatotoxicity	Furo 2 vs Top 3	333/26	0.655	-0.061	-0.015	no
Hepatotoxicity	Top 1 vs Top 2	98/28	0.004	0.389	0.047	no
Hepatotoxicity	Top 1 vs Top 3	98/26	0.004	0.400	0.078	no
Hepatotoxicity	Top 2 vs Top 3	28/26	0.568	0.104	0.016	no
Genotoxicity	Furo 1 vs Furo 2	760/333	0.017	-0.099	-0.009	no
Genotoxicity	Furo 1 vs Top 1	760/98	<0.001	-0.542	-0.037	no
Genotoxicity	Furo 1 vs Top 2	760/28	0.023	-0.276	-0.024	no
Genotoxicity	Furo 1 vs Top 3	760/26	0.278	-0.141	-0.012	no
Genotoxicity	Furo 2 vs Top 1	333/98	<0.001	-0.434	-0.026	no
Genotoxicity	Furo 2 vs Top 2	333/28	0.135	-0.193	-0.013	no
Genotoxicity	Furo 2 vs Top 3	333/26	0.771	-0.038	-0.003	no
Genotoxicity	Top 1 vs Top 2	98/28	0.145	0.206	0.006	no
Genotoxicity	Top 1 vs Top 3	98/26	0.001	0.445	0.023	no
Genotoxicity	Top 2 vs Top 3	28/26	0.419	0.148	0.012	no
Skin sensitization	Furo 1 vs Furo 2	760/333	0.002	-0.124	-0.042	no
Skin sensitization	Furo 1 vs Top 1	760/98	0.455	-0.053	-0.017	no
Skin sensitization	Furo 1 vs Top 2	760/28	<0.001	-0.495	-0.240	yes
Skin sensitization	Furo 1 vs Top 3	760/26	0.146	-0.189	-0.067	no
Skin sensitization	Furo 2 vs Top 1	333/98	0.257	0.085	0.029	no
Skin sensitization	Furo 2 vs Top 2	333/28	0.001	-0.392	-0.191	no
Skin sensitization	Furo 2 vs Top 3	333/26	0.584	-0.073	-0.023	no
Skin sensitization	Top 1 vs Top 2	98/28	<0.001	-0.483	-0.275	yes
Skin sensitization	Top 1 vs Top 3	98/26	0.361	-0.134	-0.051	no
Skin sensitization	Top 2 vs Top 3	28/26	0.122	0.280	0.132	no

Cliff's delta and HL shift are signed for group A minus group B. Strong = q_KS<0.001, q_MWU<0.001, KS D>=0.30 and axis-specific HL-shift threshold met.
